# Selective cytotoxicity of [Cu(theophylline)(H_2_O)_2_Cl_2_] complex toward cancer cells: insights from structural, spectroscopic, and molecular docking analyses

**DOI:** 10.1039/d6ra05572a

**Published:** 2026-07-31

**Authors:** Francisco N. B. Domingos, João G. de Oliveira Neto, Jéssica A. O. Rodrigues, Jailton R. Viana, Kamila R. Abreu, Aramys S. dos Reis, Mateus R. Lage, Francisco F. de Sousa, Eliana B. Souto, Adenilson O. dos Santos

**Affiliations:** a Center for Sciences of Imperatriz, Federal University of Maranhão - UFMA Imperatriz MA 65900-410 Brazil joao.gon@ufma.br; b Center for Agricultural Sciences, State University of the Tocantina Region of Maranhão – UEMASUL Imperatriz MA 65900-001 Brazil; c Institute of Exact and Natural Sciences, Federal University of Para - UFPA Belém PA 66075-110 Brazil; d UCD School of Chemical and Bioprocess Engineering, University College Dublin Belfield Dublin 4 D04 V1W8 Ireland eliana.souto@ucd.ie

## Abstract

The diaquadichlorotheophylline–copper(ii) coordination complex, [Cu(theophylline)(H_2_O)_2_Cl_2_], was synthesized *via* slow solvent evaporation and comprehensively characterized through experimental and computational approaches. X-ray powder diffraction and Rietveld refinement confirmed a triclinic crystal system (space group *P*1̄(*C*^1^_*i*_)) with pentacoordinate copper(ii) in a square-pyramidal geometry. Thermal analyses revealed thermal stability up to 358 K, with dehydration requiring 89.5 kJ mol^−1^ per H_2_O molecule. Vibrational spectroscopy (Fourier transform infrared and Raman) combined with density functional theory calculations (PBE1PBE/6-311++G(d,p)) provided suitable mode assignments and demonstrated excellent agreement between experimental and calculated spectra, including solvent effects using the IEFPCM model. Frontier molecular orbital analysis yielded the highest occupied molecular orbital-lowest unoccupied molecular orbital gaps of approximately 3.8 eV and electrophilicity indices of approximately 6.8 eV, indicating good chemical stability and biological potential. Ultraviolet-visible-near infrared spectroscopy revealed characteristic *d*–*d* transitions for pentacoordinate copper(ii) and π → π*/*n* → π* transitions from the theophylline ligand. Molecular docking predicted a strong *in silico* binding affinity to deoxyribonucleic acid (*K*_i_ = 5.85 µM, binding free energy = −7.14 kcal mol^−1^), consistent with an intercalative mode and moderate affinity to bovine serum albumin (*K*_i_ = 40.78 µM, binding free energy = −5.99 kcal mol^−1^). Cytotoxicity assays against PC-3 (prostate), MDA-MB-231 (breast), and HCT-116 (colorectal) cancer cell lines revealed dose-dependent activity with half-maximal inhibitory concentration values ranging from 2.91 to 3.77 µM and selectivity indices greater than 1 compared to the non-tumorigenic GM07429A cell line, with preferential activity against breast cancer. These results indicate that diaquadichlorotheophylline–copper(ii) is a promising anticancer candidate with favorable selectivity for malignant cells over healthy tissue, warranting further preclinical evaluation.

## Abstract 2

The diaquadichlorotheophylline–copper(ii) coordination complex, [Cu(theophylline)(H_2_O)_2_Cl_2_], was synthesized *via* slow solvent evaporation and comprehensively characterized through experimental and computational approaches.

## Graphical abstract

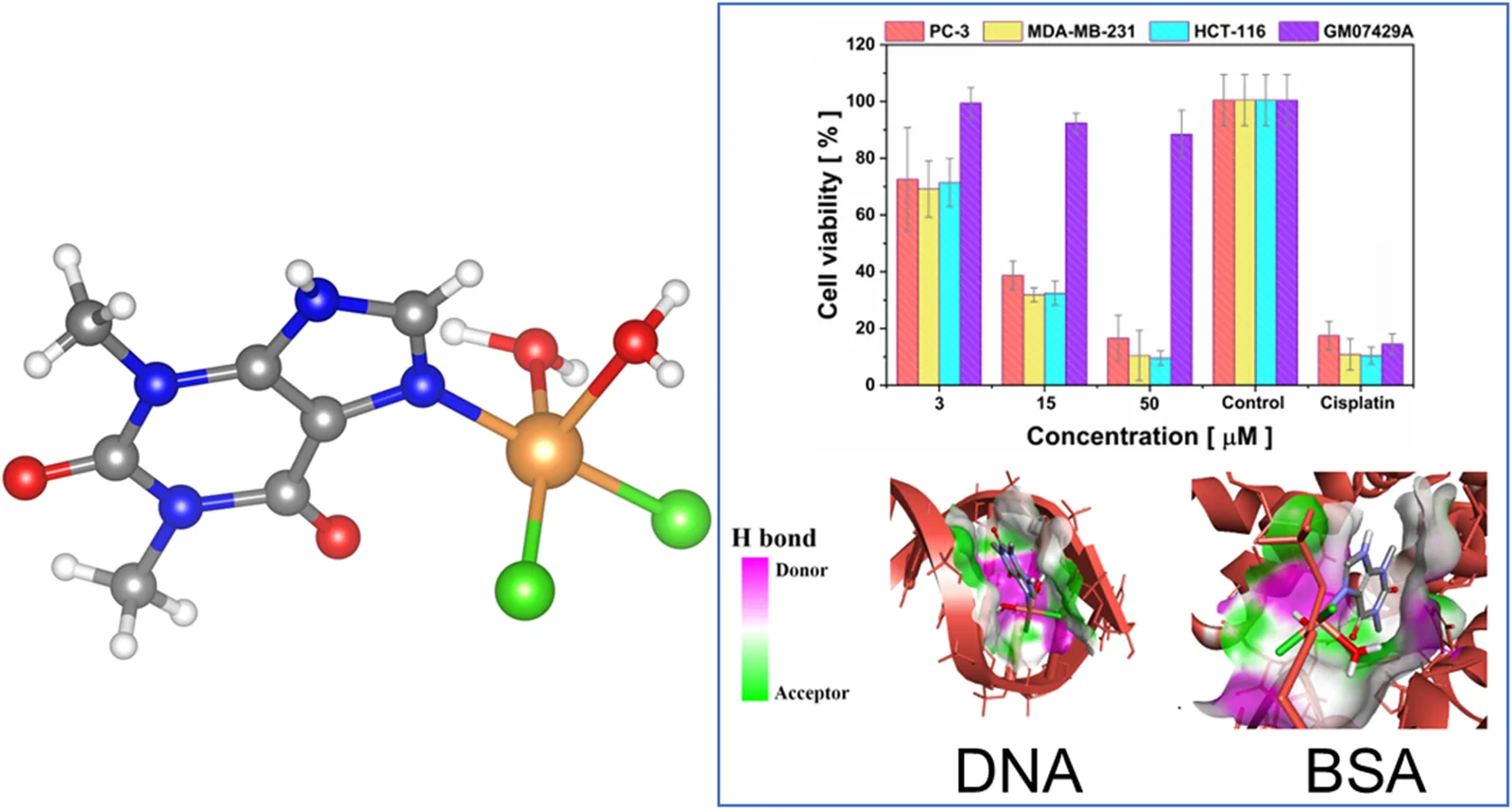

## 1 Introduction

In recent decades, metal complexes have gained prominence due to their wide range of applications in physics, chemistry, and medicine, playing a pivotal role in materials science and technology. Research on metal complexes advanced in 1965 when Rosenberg, Van Camp, and Krigas synthesized *cis*-diaminedichloroplatinum(ii) (cisplatin) and demonstrated cell growth inhibition and antitumor activity, suggesting that platinum might not be the only metal with such therapeutic properties.^1–3^ Consequently, numerous transition metal complexes have been investigated as potential innovative metal-based anticancer drugs as alternatives to platinum derivatives.^4^

Various metal–drug complexes are gradually gaining recognition, as each acts specifically in certain biological systems. Less sterically hindered metal complexes have attracted considerable attention due to their ability to interact with deoxyribonucleic acid (DNA), and these interactions can induce DNA damage in cancer cells.^5–7^ The judicious selection of appropriate ligands enables the control of physicochemical parameters. In this context, metals can act as scaffolds capable of assembling a range of suitable ligands in a unique, precise, and predictable three-dimensional (3D) geometry.^6^ In light of this, theophylline (theo) appears to be a promising candidate drug for forming chelating complexes with copper for potential applications in various fields.^8,9^

Theo is a biologically important drug that has been extensively studied since the early 1980s.^10^ It is commonly used to treat respiratory diseases, such as asthma and chronic obstructive pulmonary disease. It is a purine-derived methylxanthine, belonging to the same class as caffeine and theobromine, which constitute an important family of bronchodilators.^11,12^ The chemical structure of theo comprises two fused aromatic rings with two methyl groups attached to nitrogen atoms, thereby blocking metal cation coordination at these positions and leaving an available coordination site at the remaining nitrogen atom.^13,14^ Consequently, the preparation of metal–theo complexes can enhance therapeutic activities and contribute significantly to advancements in materials science.^15^

Theo complexes with transition metals have been reported with promising biological activities. Gacki *et al.*^16^ synthesized [Mn(theo)_2_(H_2_O)_4_], [Co(theo)_2_(H_2_O)_4_], and [Ni(theo)_2_(H_2_O)_4_]. All investigated complexes exhibited significant radical scavenging activity (RSA), with the first two displaying the highest activity, following the order Mn > Co > Ni. Additionally, [Co(theo)_2_(H_2_O)_4_] showed moderate antimicrobial activity against Gram-positive bacteria with a minimum inhibitory concentration (MIC) range of 125–500 mg L^−1^, while [Mn(theo)_2_(H_2_O)_4_] showed slight bioactivity against *Staphylococcus aureus* and *Micrococcus luteus* (MIC range of 500–1000 mg L^−1^).

Moreover, transition metal complexes with theo have attracted increasing interest due to their promising biological activities, with copper–theo complexes being particularly noteworthy. Gordon *et al.*^17^ investigated several complexes, including [Cu(theo)_2_(H_2_O)_3_]·2H_2_O, which exhibited dose-dependent inhibition of pancreatic cancer cell lines. Despite the relevance of these compounds, it should be noted that limited information is available in literature, and some complexes have only been structurally characterized without further biological evaluation. A 2 : 1 theophylline : Cu(ii) complex has been previously reported.^18^ In the present work, we focus on the 1 : 1 stoichiometry, studying the [Cu(theo)(H_2_O)_2_Cl_2_] complex, whose combined structural, vibrational, electronic and biological characterization has not been described yet.

Although the [Cu(theo)(H_2_O)_2_Cl_2_] complex was first synthesized and structurally characterized by single-crystal X-ray diffraction by Cingi *et al.* in 1979,^19^ its physicochemical properties, and particularly, its biological potential have remained unexplored to date. To the best of our knowledge, no thermal, vibrational, electronic, computational, or biological data have been reported for this compound. Therefore, the present study does not aim to redetermine its crystal structure. Instead, it builds upon the previously reported structural model to provide a comprehensive, multi-technique characterization and to assess its anticancer potential for the first time.

Herein, the phase purity of the material was confirmed by X-ray powder diffraction (XRPD) and Rietveld refinement; the thermal decomposition pathway and the dehydration energetics of the coordinated H_2_O molecules were quantified by thermogravimetry (TG), differential thermal analysis (DTA), and differential scanning calorimetry (DSC); and the vibrational modes were assigned by combining Fourier transform infrared spectroscopy (FT-IR) and Raman spectroscopy with density functional theory (DFT) calculations under explicit solvation. In addition, DFT was further employed to describe the electronic structure through frontier molecular orbital analysis, chemical reactivity descriptors, and molecular electrostatic potential mapping. Complementarily, the optical properties were probed experimentally by ultraviolet-visible-near infrared (UV-Vis-NIR) spectroscopy, and the observed bands were interpreted within the framework of ligand-field theory. Finally, molecular docking against DNA and bovine serum albumin (BSA), together with *in vitro* cytotoxicity assays on PC-3 (prostate), MDA-MB-231 (breast), and HCT-116 (colorectal) tumor cell lines, as well as the non-tumorigenic GM07429A line, revealed a selective anticancer profile not previously described for this complex.

## 2 Materials and methods

### 2.1 Crystals growth

The [Cu(theo)(H_2_O)_2_Cl_2_] complex was synthesized *via* the slow solvent evaporation method. Two starting materials were used, *i.e.*, theo (Sigma-Aldrich, ≥99% purity) and copper(ii) chloride dihydrate (Sigma-Aldrich, ≥99% purity), in a molar ratio of 2 : 1 (0.5405 g : 0.2257 g). These precursor compounds were weighed and dissolved in 60 mL of ethanol (Sigma-Aldrich - selected for its ability to dissolve both precursors and its suitable volatility for controlled slow-evaporation crystal growth), followed by magnetic stirring at 360 rpm and 313 K for 180 min. The solution was subsequently filtered, covered with perforated plastic film, and kept in an oven at 308 K for seven days to allow slow solvent evaporation and crystal formation.

### 2.2 Characterization techniques

XRPD was performed using a PANalytical Empyrean diffractometer equipped with CuKα radiation (*λ* = 1.5418 Å), Bragg–Brentano geometry, and a pyrolytic graphite monochromator. The analysis was conducted at room temperature (298 K) with an angular step size of 0.02° and an acquisition time of 2 s per step, covering an angular range of 8–50° (2*θ*). The XRPD pattern was refined by the Rietveld method using the GSAS-EXPGUI package,^20^ with parameters taken from the literature^19^ and applying the standard statistical weighting scheme *w*_i_ = 1/*σ*^2^(*y*_i_) based on Poisson counting statistics.

TG-DTA measurements were simultaneously performed from 303 to 1100 K using a Shimadzu DTG-60 thermogravimetric analyzer. The differential scanning calorimetry (DSC) curve was recorded from 303 to 770 K using a Shimadzu DSC-60 instrument. Approximately 4.0 mg of sample were analyzed under a nitrogen atmosphere with a flow rate of 100 mL min^−1^ and a heating rate of 10 K min^−1^, using an open aluminum crucible.

The FT-IR spectrum was obtained using a Bruker Vertex 70 V spectrometer with the KBr pellet technique. The analysis was conducted using three excitation sources and an InGaAs detector, as well as a DLaTGS detector, and averaged 32 scans with a spectral resolution of 4 cm^−1^.

The Raman spectrum was recorded using a Horiba LabRAM Evolution spectrometer equipped with a Peltier-cooled charge-coupled device (CCD) detector. The excitation source was a green laser (*λ* = 514 nm) with a power of 2.90 mW. The spectral range analyzed was 30–3600 cm^−1^, with five accumulations and a spectral resolution of 4 cm^−1^.

UV-Vis-NIR spectroscopy measurements were performed using a Shimadzu UV-Vis-NIR spectrophotometer (model UV-1900). The analyses were conducted in the 190–1100 nm range with a dual-beam configuration using deuterium and tungsten lamps, employing quartz cuvettes with a 0.1 cm optical path length.

### 2.3 DFT study

The calculations were performed using the Gaussian 16 software package,^21^ based on the geometry from the CIF code 1101052 for the [Cu(theo)(H_2_O)_2_Cl_2_] complex,^19^ obtained from the Cambridge Crystallographic Data Centre (CCDC). The DFT functional PBE1PBE,^22^ widely employed in the study of coordination compounds, was selected as it provides results in good agreement with experimental data.^23,24^ The 6-311++G(d,p) basis set was applied to C, N, H, O, and Cl atoms, while the Stuttgart-Dresden (SDD) pseudopotential was used for the core electrons of the Cu atom and its associated basis functions were used for the valence electrons.^25,26^ Solvation effects were considered using the integral equation formalism of the polarizable continuum model (IEFPCM) for ethanol and water, in addition to the calculations performed under vacuum conditions. The computational output files were processed using the ChemCraft graphical interface software, confirming the absence of imaginary frequencies, thereby verifying that the optimized structures correspond to a true minima on the potential energy surfaces. The calculated vibrational modes were analyzed using the vibAnalysis software, which applies Bayesian regression to determine the potential energy distribution (PED) of the vibrational modes.^27^ The calculated spectra were corrected using a scaling factor of 0.9594.^28^ Finally, molecular electrostatic potential (MEP) values were computed using the Multiwfn software package.^29^ All calculations were performed for the neutral complex in its doublet spin state, consistent with the *d*^9^ electronic configuration of the copper(ii) center. Accordingly, an unrestricted DFT formalism (UDFT; UPBE1PBE) was employed.

### 2.4 Molecular docking

Molecular docking studies of the [Cu(theo)(H_2_O)_2_Cl_2_] complex with DNA and BSA macromolecules were performed using AutoDock Vina, with the assistance of AutoDock Tools (version 1.5.7).^30^ The 3D structural coordinates of the DNA dodecamer and BSA were obtained from the Protein Data Bank (PDB) under PDB IDs 1BNA and 4F5S, respectively.^31,32^ The optimized structure of the complex, obtained *via* DFT calculations as described in Section 2.3, was converted to PDB format for docking studies. The macromolecular structures were prepared by removing heteroatoms and water molecules, followed by the addition of Kollman charges and polar hydrogens. Subsequently, PDBQT files for both the macromolecules and the complex were generated. For DNA (PDB 1BNA), a grid box of 60 × 60 × 60 points with 0.375 Å spacing was used. For BSA, as a single AutoGrid4 map cannot enclose the entire protein at the standard 0.375 Å resolution (126-point per-dimension limit), the largest grid box permitted at this resolution was employed: 126 × 126 × 126 points with a grid spacing of 0.375 Å, centered on the macromolecule to sample its principal binding region. The Lamarckian Genetic Algorithm was employed with 100 independent runs.^33^ To assess the binding affinity of the complex to its targets, the following parameters were calculated: inhibition constant (*K*_i_), binding free energy (Δ*G*_bind_), estimated total intermolecular energy comprising van der Waals (vdW) interactions, hydrogen bonding (H-bond), and desolvation energy.

### 2.5 Cell viability

The human tumor cell lines PC-3 (prostate), MDA-MB-231 (breast), and HCT-116 (colorectal), along with the non-tumorigenic human cell line GM07429A (lung fibroblast), all obtained from American Type Culture Collection (ATCC®), were used to evaluate the cytotoxicity of the [Cu(theo)(H_2_O)_2_Cl_2_] complex. Each cell line was cultured in Dulbecco's Modified Eagle's Medium (DMEM) supplemented with 10% fetal bovine serum and 1% antibiotic-antimycotic solution (10 000 U mL^−1^ penicillin, 10 000 µg mL^−1^ streptomycin, and 25 µg mL^−1^ amphotericin B), Gibco. The cultures were maintained at 310 K in a humidified atmosphere containing 5% CO_2_. For the experiments, 1 × 10^4^ cells per well were seeded in 96-well plates and incubated for 24 h. The cells were then treated with the [Cu(theo)(H_2_O)_2_Cl_2_] complex at concentrations of 3, 15, and 50 µM (100 µL per well) and cisplatin (5 mM) as a reference drug (positive control) for 24 h. Untreated cells were used as the negative control. Cytotoxicity was assessed using the (3-(4,5-dimethylthiazol-2-yl)-2,5-diphenyltetrazolium bromide (MTT)) assay by adding 10 µL of MTT reagent to each well after treatment. The cells were then incubated in the dark at 37 °C in a 5% CO_2_ atmosphere for 3 h. Following the formation of formazan crystal, the MTT-containing medium was removed, and 100 µL of absolute ethanol was added to dissolve the formazan crystals. Absorbance was measured using a microplate spectrophotometer (Epoch, BioTek Instruments) at 570 nm. IC_50_ value (the concentration required to inhibit 50% of cell viability) was determined by nonlinear regression (log[inhibitor] *vs.* normalized response) using OriginPro 2016, and is reported as mean ± standard deviation (SD) with the corresponding 95% confidence interval. To assess the selectivity of the [Cu(theo)(H_2_O)_2_Cl_2_] complex, the selectivity index (SI) was calculated as follows:
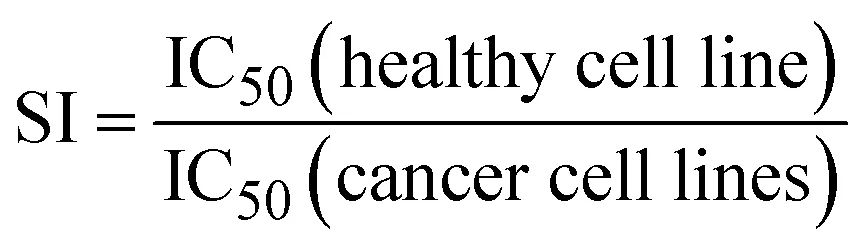


A SI value > 1 indicates that the complex exhibits selectivity for cancer cells over non-tumorigenic cells.^34^ All treatments were performed in three technical replicates (wells) per concentration, and each experiment was independently repeated in three biological replicates (*n* = 3). Data are expressed as mean ± standard deviation (SD). Statistical significance was further assessed by one-way ANOVA followed by Dunnett's post-hoc test (*p* < 0.05).

## 3 Results and discussion

### 3.1 Structural analysis *via* XRPD and Rietveld refinement

**Fig. 1:**
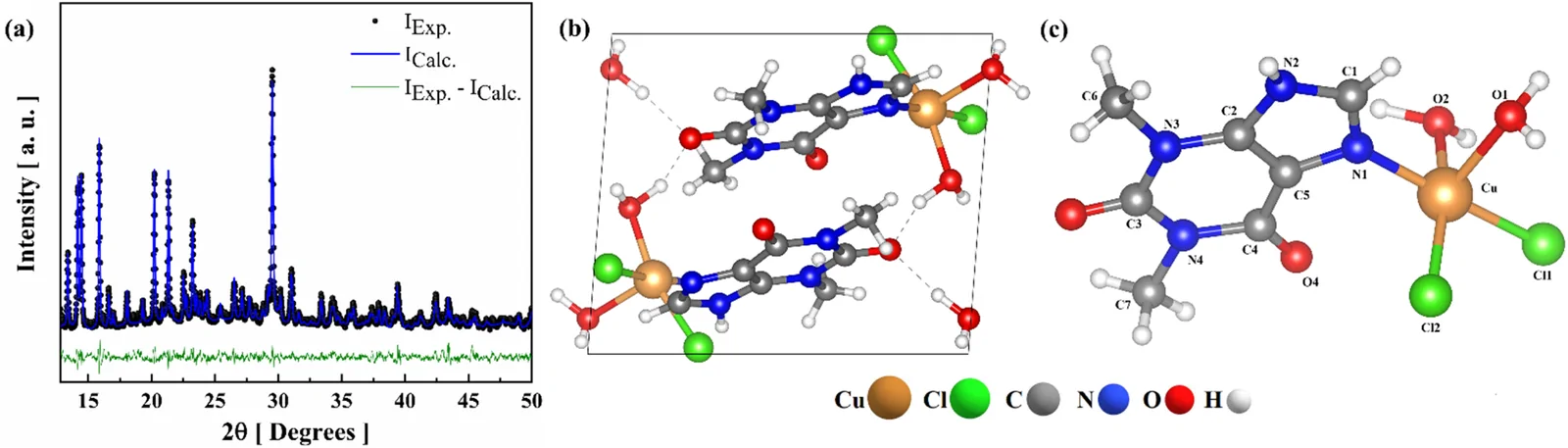
(a) XRPD pattern and Rietveld refinement for the [Cu(theo)(H_2_O)_2_Cl_2_] complex. (b) Unit cell viewed along the *c*-axis. (c) Asymmetric unit of the complex with labels.

The crystal structure of the [Cu(theo)(H_2_O)_2_Cl_2_] complex was identified by XRPD and Rietveld refinement, as shown in Fig. 1(a). The experimental diffractogram (black dots) was compared with the calculated pattern (blue line) obtained from literature data.^19^ At room temperature (298 K), the sample crystallizes in the triclinic system with space group *P*1̄ (*C*^1^_*i*_) and two formula units per unit cell (*Z* = 2) (Fig. 1(b)). The refined lattice parameters were *a* = 9.926(5) Å, *b* = 9.991(5) Å, *c* = 7.478(4) Å, and *α* = 123.1(4)°, *β* = 94.4(4)°, *γ* = 83.8(5)°. These values are consistent with those reported in the literature,^19^ as shown in Table 1. The refinement reliability factors were *R*_wp_ = 8.70%, *R*_p_ = 6.09%, and *S* = 1.81. For powder Rietveld refinements, an S value in this range, combined with the low-profile residuals, confirms a good agreement between the observed and calculated diffraction patterns, thereby validating the quality of the refinement and the phase purity of the sample. The [Cu(theo)(H_2_O)_2_Cl_2_] complex was synthesized in several independent batches, and the phase purity of each was confirmed by XRPD and Rietveld refinement, which consistently reproduced the same single-phase pattern, attesting to the reproducibility of the preparation. The asymmetric unit of the [Cu(theo)(H_2_O)_2_Cl_2_] complex is shown in Fig. 1(c). The Cu^2+^ ion exhibits pentacoordination in a square-pyramidal geometry, with a coordination sphere consisting of one theo molecule coordinated through a nitrogen (N) atom, two chloride (Cl^−^) ions, and two water (H_2_O) molecules.

**Table 1:** Crystallographic and refinement parameters of the [Cu(theo)(H_2_O)_2_Cl_2_] complex

Parameter	Literature (Cingi, 1979)	Present study
Empirical formula	C_7_H_12_CuCl_2_N_4_O_4_	C_7_H_12_CuCl_2_N_4_O_4_
Formula weight [g mol^−1^]	350.65	350.65
Temperature [K]	298	298
Radiation/wavelength [Å]	MoKα (0.71069)	CuKα (1.5418)
Crystal system	Triclinic	Triclinic
Space group	*P*1̄ (*C*^1^_*i*_)	*P*1̄ (*C*^1^_*i*_)
*a* [Å]	9.930(8)	9.926(5)
*b* [Å]	9.987(8)	9.991(5)
*c* [Å]	7.483(6)	7.478(4)
*α* [°]	123.2(1)	123.1(4)
*β* [°]	94.5(1)	94.4(4)
*γ* [°]	83.9(1)	83.8(5)
*Z*	2	2
*V* [Å^3^]	617(1)	617.2(3)
Calc. density [g cm^−3^]	1.887	1.887
Refinement method	Single-crystal (*F*^2^)	Rietveld (powder)
2*θ* range [°]	—	8–50
Weighting scheme	—	*w*_i_ = 1/*σ*^2^(*y*_i_)
*R*_wp_ [%]	—	8.70
*R*_p_ [%]	—	6.09
*S*	—	1.81
CCDC number	1101052	—

The copper(ii) center adopts a distorted square-pyramidal geometry, as confirmed by the Addison structural parameter *τ*_5_ = 0.19 (*τ*_5_ < 0.5 indicates a square-pyramidal arrangement, whereas *τ*_5_→ 1 corresponds to a trigonal-bipyramidal one). The basal plane is defined by the theo nitrogen atom (Cu–N1 = 1.98 Å), one chloride ligand (Cu–Cl2 = 2.27 Å), the second chloride (Cu–Cl1 = 2.29 Å), and one H_2_O molecule (Cu–O2 = 2.02 Å), while the apical position is occupied by the second H_2_O molecule at a longer distance (Cu–O1 = 2.29 Å), consistent with the axial elongation typically observed for Jahn–Teller active *d*^9^ Cu^2+^ centers. The two *trans* basal angles, N1–Cu–Cl1 = 175.1° and Cl2–Cu–O2 = 163.3°, deviate appreciably from linearity, reflecting the distortion of the coordination polyhedron.

The coordination bond lengths of [Cu(theo)(H_2_O)_2_Cl_2_] agree well with those reported for structurally analogous square-pyramidal copper(ii) complexes. The equatorial Cu–N1 distance (1.98 Å) lies within the typical range for Cu–N(aromatic) bonds in copper(ii)–theo and related N-donor systems (1.97–2.05 Å).^17^ The equatorial Cu–Cl bonds (2.27 and 2.29 Å) are consistent with terminal Cu–Cl distances reported for square-pyramidal copper(ii) chloride complexes (2.20–2.30 Å).^35^ Notably, the apical Cu–O4 bond (2.29 Å) is markedly longer than the equatorial Cu–O5 bond (2.02 Å), in line with the general trend that apical Cu–L distances exceed the equatorial ones in five-coordinate copper(ii), where the apical site is preferentially occupied by O-donor ligands, as a consequence of Jahn–Teller elongation at the *d*^9^ center.^36^ Comparable axial Cu–OH_2_ distances of 2.31–2.35 Å have been reported for related aqua–copper(ii) complexes,^17,35^ confirming that the present data are fully consistent with the literature.

The [Cu(theo)(H_2_O)_2_Cl_2_] crystal packing is stabilized by an extended 3D lattice of hydrogen bonds. The coordinated H_2_O molecules act as hydrogen-bond donors toward the carbonyl oxygen atoms of neighboring theo ligands (O–H⋯O = 2.783 and 2.907 Å) and toward the chloride ligands (O–H⋯Cl = 3.181 and 3.256 Å), while the N–H group of the theo ring donates a hydrogen bond to a chloride ligand of an adjacent complex (N–H⋯Cl = 3.138 Å). This combination of O–H⋯O, O–H⋯Cl, and N–H⋯Cl interactions link the mononuclear units into a stable supramolecular framework.

The theo ligand retains an essentially planar fused-ring conformation upon coordination, with a maximum deviation of only 0.027 Å from the mean plane defined by the nine ring atoms. Within the five-membered imidazole ring, the torsion angles are all below 1° (*e.g.*, N1–C1–N2–C2 = 0.3°), while the six-membered pyrimidinedione ring shows a very slight puckering, with the largest torsion angles of −5.1° (C4–C5–C2–N3) and +4.3° (N4–C4–C5–C2). Both carbonyl groups lie close to the ring plane, as indicated by the O–C–N–C torsion angles near 180° (177.6° and 178.1°). Notably, the copper(ii) center is nearly coplanar with the theo ring, as evidenced by the Cu–N1–C1–N2 (170.5°) and Cu–N1–C5–C2 (−171.6°) torsion angles, which approach the ideal value of 180°.

### 3.2 TG-DTA and DSC thermal analyses

**Fig. 2:**
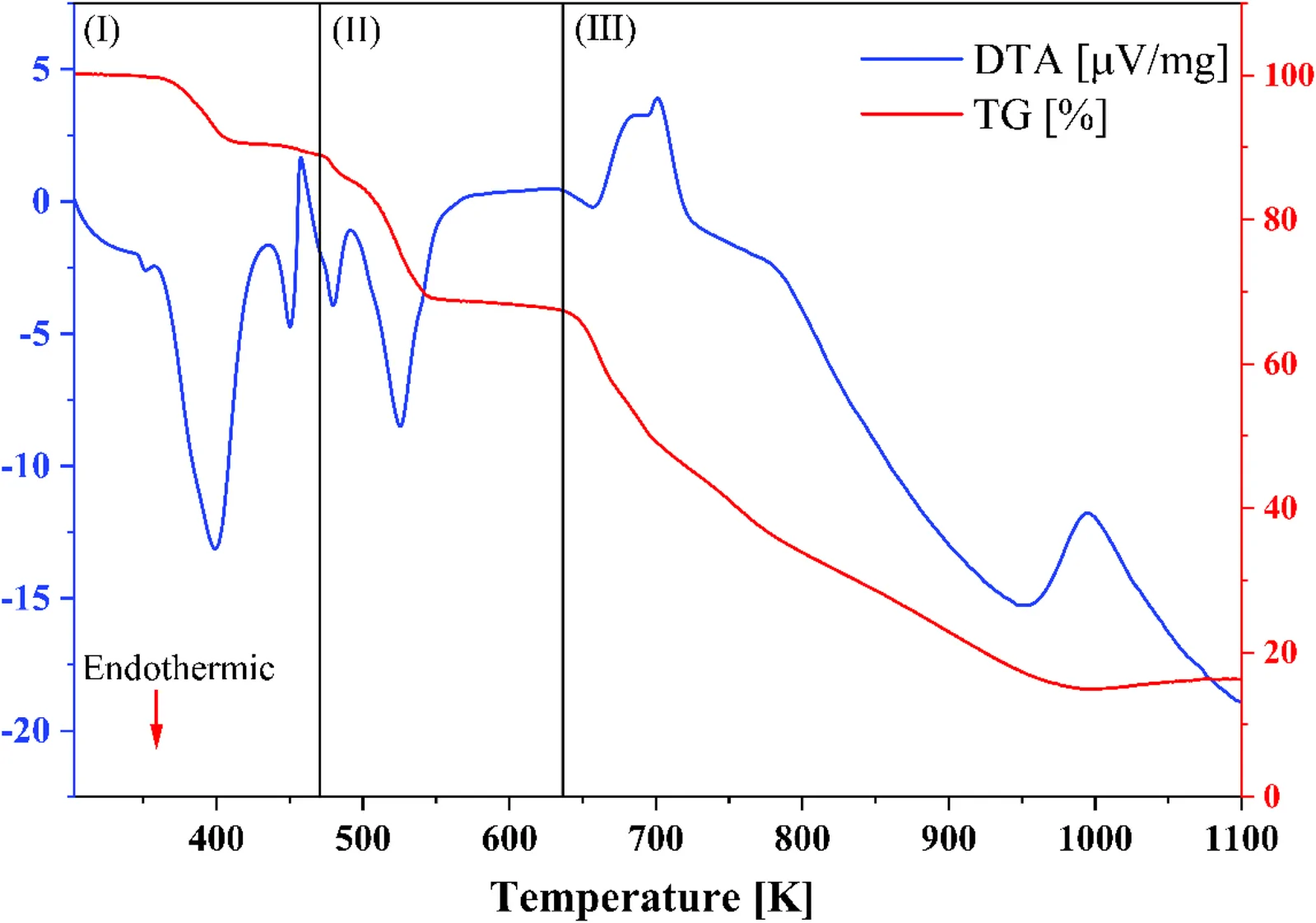
TG-DTA thermogram of the [Cu(theo)(H_2_O)_2_Cl_2_] complex.

The TG-DTA thermogram of the [Cu(theo)(H_2_O)_2_Cl_2_] complex, presented in Fig. 2, exhibits three distinct regions of thermal events. Region (I) corresponds to the dehydration process involving the loss of two coordinated H_2_O molecules, observed in the TG curve between 358 and 454 K, with a mass loss of 9.69% (33.96 g mol^−1^). The removal of H_2_O molecules corresponds to endothermic events in the DTA curve, with peaks at 399 and 450 K. Additionally, an exothermic event is observed in the DTA curve between 455 and 468 K (peak at 457 K), which is likewise reproduced in the DSC trace (454–465 K, peak at 456 K). This thermal event is not accompanied by any mass loss in the TG curve and occurs after the completion of dehydration (454 K) but before the onset of ligand decomposition (470 K). An exothermic signal without an associated mass change is characteristic of a solid-state phase change. Because dehydration takes place well above the boiling point of water,^37^ the initially formed anhydrous phase is likely generated in a metastable, poorly ordered state and subsequently undergoes an exothermic recrystallization into a more stable anhydrous form. Such dehydration-induced structural reorganizations are well documented for hydrated complexes, including copper(ii) systems in which the removal of coordinated water triggers solid-state phase transformations.^38,39^ A definitive crystallographic identification of this transition, however, would require temperature-resolved (*in situ*) XRPD, which is proposed for future investigation.

Region (II), in the temperature range of 470–636 K, corresponds to the partial decomposition of the organic moiety of the theo ligand. TG analysis shows a mass loss of 22.15% (78.15 g mol^−1^). Within this temperature range, two endothermic events are recorded in the DTA curve: one occurring from 470 to 488 K with a peak at 481 K, and another from 494 to 570 K with a peak at 525 K. Notably, the theo ligand does not undergo complete decomposition in this range, as its molecular weight is 180.16 g mol^−1^. This result indicates that coordination to a metal ion increases the thermal stability of the organic ligand, requiring greater energy for its decomposition.

In the third region (III), between 635 and 1100 K, TG analysis reveals the decomposition of the remaining theo fragments and Cl^−^ ions, resulting in a mass loss of 52.36%. Endothermic peaks are observed in the DTA curve at lower temperatures, while exothermic peaks at higher temperatures may be associated with the release of gases during the combustion of matter. At 1100 K, a residual mass of 16.34% was identified, corresponding to the copper content in the oxide form. All thermal events observed in the TG-DTA curves are summarized in Table 2.

**Table 2:** Thermal events of the [Cu(theo)(H_2_O)_2_Cl_2_] complex observed in TG-DTA and DSC curves^a^

Fragments	Region	Temperature ranges [K]	DTA	DSC	TG	Mass loss [mg]	Molar mass [g mol^−1^]	
			Temperature [K]		Mass loss [%]			
							Exp	*Cal*
2H_2_O	I	358–458	399 (↓)	384 (↓)	9.69	0.394	34.18	32.00
			450 (↓)	449 (↓)				
			458 (↑)	456 (↑)				
C_7_H_8_N_4_O_2_	II	458–635	481 (↓)	478 (↓)	21.61	0.877	76.24	180.16
			525 (↓)	407 (↓)				
				527 (↓)				
C_7_H_8_N_4_O_2_Cl_2_	III	635–1100	657 (↓)	725 (↑)	52.36	2.126	184.74	251.00
							
			702 (↑)					
			952 (↓)					
Total molecular weight							352.83	350.50

a Thermal events identified in the DTA and DSC curves: ↓ - endothermic; ↑ - exothermic.

**Fig. 3:**
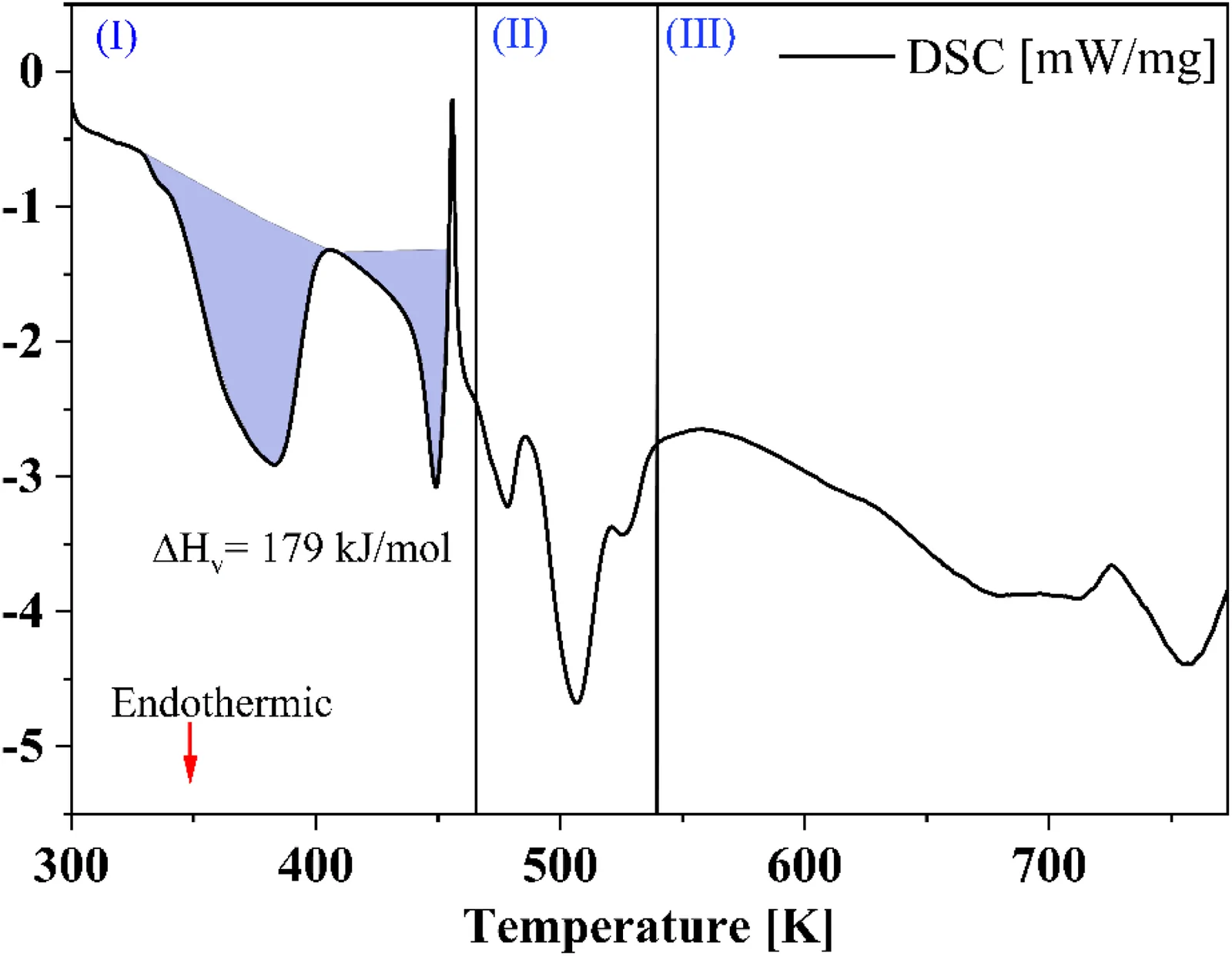
DSC curve of the [Cu(theo)(H_2_O)_2_Cl_2_] complex.

Fig. 3 presents the DSC results, which are consistent with the DTA events observed. In Region (I), two endothermic events were recorded, corresponding to the evaporation of the two coordination H_2_O molecules: the first occurring between 337 K and 404 K, with a peak at 384 K, and the second between 408 K and 454 K, with a maximum at 449 K. Additionally, in the first region, an exothermic event was observed from 454 K to 465 K, with a peak at 456 K. The dehydration enthalpy of the complex, determined from the two endothermic peaks highlighted in Fig. 3, is 179 kJ mol^−1^. The removal of each H_2_O molecule in the [Cu(theo)(H_2_O)_2_Cl_2_] complex requires 89.5 kJ mol^−1^, which is approximately twice the enthalpy of dehydration of liquid water (∼40 kJ mol^−1^).^37^ This energy difference of 49.5 kJ mol^−1^ between water dehydration and the dehydration of a coordinated H_2_O molecule can be attributed to the metal–ligand bond strength and intermolecular interactions within the crystal lattice.

Regions (II) and (III) correspond to the decomposition of the organic ligand and Cl^−^ ions. The specific temperatures of the thermal events observed in these regions are provided in Table 2. These analyses indicate that the [Cu(theo)(H_2_O)_2_Cl_2_] complex exhibits thermal stability up to 358 K, consistent with the thermal behavior of similar coordination compounds.^24,40^

### 3.3 DFT study

#### 3.3.1 Geometry optimization

**Fig. 4:**
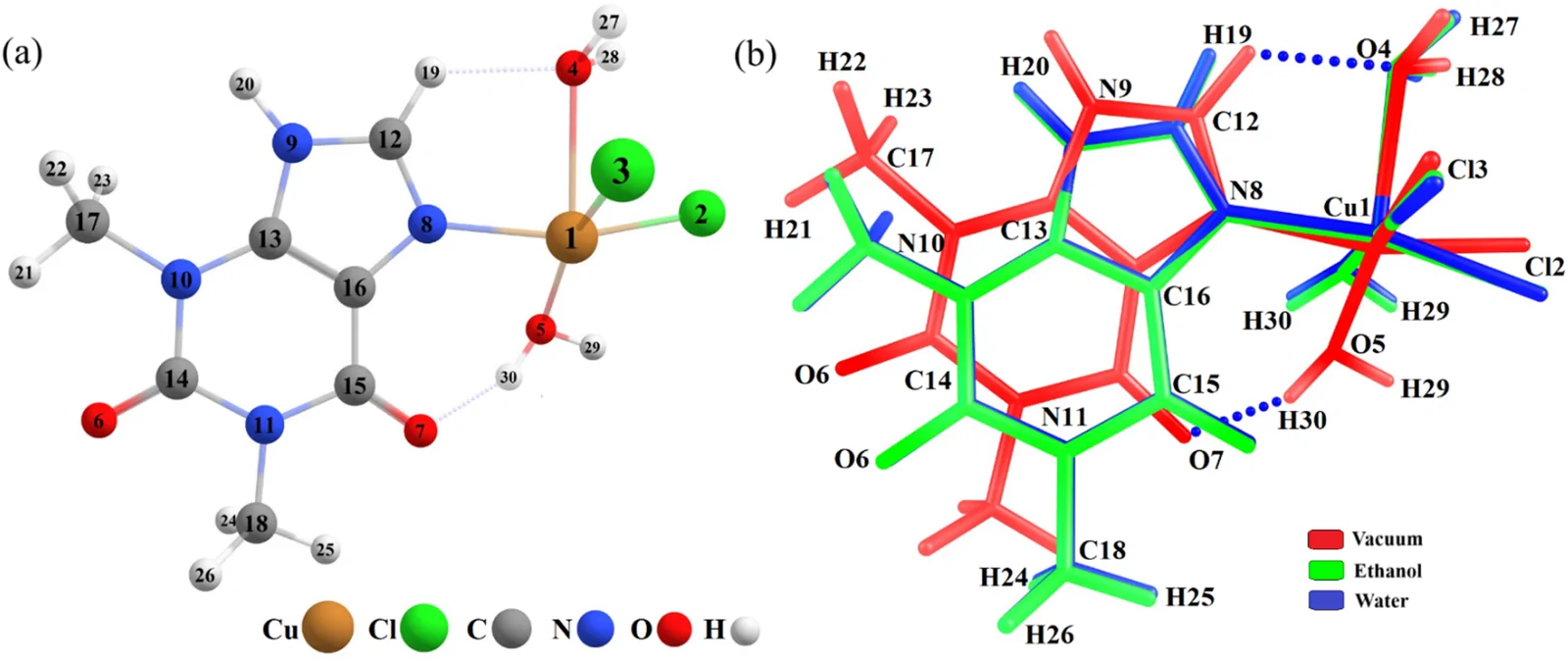
Optimized geometry of the [Cu(theo)(H_2_O)_2_Cl_2_] complex: (a) in vacuum and (b) structural overlay showing optimized geometries in vacuum (red), ethanol (green), and water (blue).

The optimized geometry of the [Cu(theo)(H_2_O)_2_Cl_2_] complex was obtained under vacuum conditions (Fig. 4(a)) and in ethanol and water solvents using DFT calculations. Fig. 4(b) compares the geometries obtained in vacuum, ethanol, and water. Based on the optimized geometry of the complex, the bond lengths (Å) and bond angles (°) were analyzed and compared with experimental crystallographic data available in the literature.^19^Table 3 presents the bond lengths and bond angles within the coordination sphere of the complex.

**Table 3:** Geometric parameters of the [Cu(theo)(H_2_O)_2_Cl_2_] complex: bond lengths [Å], bond angles [°], and RMSD values

	Exp [19]	Ethanol	Water	Vacuum	RMSD ethanol	RMSD water	RMSD vacuum
**Bond type [Å]**							
Cu–N8	1.98	2.00	2.00	2.05	0.012	0.012	0.034
Cu–O5	2.02	2.09	2.09	2.05	0.035	0.035	0.015
Cu–Cl2	2.27	2.27	2.27	2.21	0.004	0.001	0.030
Cu–Cl3	2.29	2.27	2.27	2.22	0.008	0.008	0.033
Cu–O4	2.29	2.34	2.33	2.35	0.027	0.021	0.032

**Bond angle [°]**							
N8–Cu–O5	88.0	86.7	86.7	87.6	0.030	0.030	0.032
N8–Cu–Cl3	91.5	92.0	92.0	92.5	0.014	0.015	0.040
Cl3–Cu–Cl2	92.5	96.3	96.1	101.8	0.047	0.045	0.103
Cl1–Cu–O5	87.3	84.4	84.5	89.0	0.038	0.038	0.032
Cl3–Cu–O5	163.3	177.4	177.6	152.2	0.129	0.130	0.110
N8–Cu–Cl2	175.1	167.3	166.3	154.6	0.068	0.076	0.110
O4–Cu–N8	85.5	89.1	89.0	84.2	0.064	0.059	0.037
O4–Cu–O5	93.3	90.5	86.8	134.0	0.039	0.071	0.475
O4–Cu–Cl2	96.3	100.4	100.8	80.0	0.062	0.069	0.219
O4–Cu–Cl3	103.0	91.7	95.2	73.3	0.141	0.096	0.398
C12–N8–Cu	130.5	128.6	128.7	119.1	0.018	0.018	0.085
C16–N8–Cu	124.9	124.7	124.6	132.9	0.010	0.010	0.078

The calculated bond lengths and angles for Cu–N8, Cu–O5, Cu–Cl2, Cu–Cl3, and Cu–O4 show good agreement with the experimental data.^19^ The largest discrepancy in bond length was 0.07 Å for the Cu–O5 bond in both ethanol and water solvents, with a root mean square deviation (RMSD) value of 0.035 Å. The smallest deviation was observed for the Cu–Cl2 bond, where the calculated values in ethanol and water closely matched the experimental values, with RMSD values of 0.004 and 0.001 Å, respectively.

Regarding the bond angles in the coordination sphere, the greatest deviation between experimental and calculated values occurred for the O4–Cu–O5 angle, with a difference of 40.7° in vacuum and a RMSD of 0.475°. In ethanol, the largest deviation was observed for the Cl3–Cu–O4 angle (14.1°, RMSD = 0.129°), while in water, it was for the Cl3–Cu–O5 angle (14.3°, RMSD = 0.130°). The smallest deviation in bond angles was observed for the N8–Cu–O4 angle, with differences of 1.3°, 1.3°, and 0.4° and RMSD values of 0.030°, 0.030°, and 0.032° for ethanol, water, and vacuum, respectively.

The largest angular deviation in the solvated models is the *trans* Cl3–Cu–O5 angle (163.3° experimental *vs.* 177.6° in water; RMSD = 0.130°), a soft bending coordinate involving a coordinated H_2_O molecule. In the crystal, it is bent away from linearity by intermolecular hydrogen bonding (O–H⋯O and O–H⋯Cl) and 3D packing, whereas the geometry of the isolated complex, which reproduces only the bulk solvent dielectric and not these specific interactions, relaxes toward a near-linear *trans* arrangement, accounting for the larger deviation.

In the calculations performed under vacuum conditions, it is observed that atoms H19 and O4 move closer together, reducing their distance to 1.99 Å, whereas under solvation conditions, the distance increases to 2.52 Å. A similar effect is observed for H30 and O7, where the distance is 1.76 Å under vacuum but increases to 3.31 Å in ethanol and 3.35 Å in water. These results suggest the formation of intramolecular hydrogen bonding interactions (H19⋯O4 and H30⋯O7) under vacuum conditions, which are disrupted by solvation effects, highlighting the influence of the solvent environment on the molecular geometry of the complex.^41^

Overall, the calculated bond lengths and bond angles in ethanol and water showed good correlation with experimental crystallographic data, as confirmed by the low RMSD values presented in Table 3. In the vacuum calculation, a slight difference in the spatial arrangement of the complex is observed, as shown in Fig. 4(b). However, this did not result in significant deviations in bond lengths and angles. Additionally, these results are consistent with previous studies on copper(ii) complexes that employed the same hybrid PBE0 (PBE1PBE) functional and comparable basis sets/effective-core potentials to reproduce the experimental geometry as well as the vibrational and electronic properties of similar systems.^42,43^

As discussed, the geometry optimizations calculations were performed at the PBE1PBE level without empirical dispersion correction, as the first coordination sphere already reproduces the X-ray geometry with low RMSD values (Table 3), confirming that the metal–ligand bonding is governed mainly by coordinate and electrostatic interactions rather than long-range dispersion. As the isolated-complex/IEFPCM model does not include intermolecular packing, dispersion-corrected schemes such as Grimme's D3(BJ) would be required to describe such weak non-covalent contacts,^44,45^ which may be addressed in future work.

#### 3.3.2 Frontier molecular orbitals, chemical reactivity descriptors, and MEP

The energy values of the highest occupied molecular orbital (HOMO) and lowest unoccupied molecular orbital (LUMO) were obtained, allowing the calculation of chemical reactivity descriptors for the complex, namely, HOMO–LUMO gap (HLG), ionization potential (IP), electron affinity (EA), electronegativity (*χ*), chemical potential (*µ*), chemical hardness (*η*), softness (*ς*), and electrophilicity index (*ω*). These descriptors were derived using mathematical relationships based on the HOMO and LUMO energy values. Table 4 presents the equations and calculated values for the [Cu(theo)(H_2_O)_2_Cl_2_] complex.

**Table 4:** Global reactivity descriptors of the complex [Cu(theo)(H_2_O)_2_Cl_2_], calculated in ethanol, water and vacuum

Reactivity descriptors	Mathematical relationship	Energy [eV] ethanol	Energy [eV] water	Energy [eV] vacuum
HOMO	—	−7.02	−6.99	−7.08
LUMO	—	−3.19	−3.20	−3.21
HLG	HLG = LUMO − HOMO	3.83	3.79	3.87
IP	IP = – HOMO	7.02	6.99	7.08
EA	EA = – LUMO	3.19	3.20	3.21
*χ*	*χ* = −1/2 (LUMO + HOMO)	5.10	5.10	5.15
*µ*	*µ* = 1/2 (LUMO + HOMO)	−5.10	−5.10	−5.15
*η*	*η* = 1/2 (LUMO − HOMO)	1.91	1.90	1.94
*ς* a	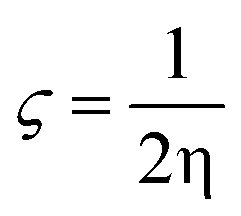	0.26	0.26	0.26
*ω*	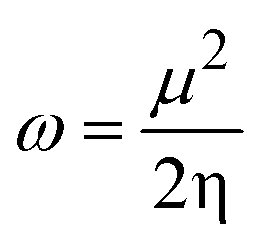	6.80	6.84	6.84

a *eV^−1^.

**Fig. 5:**
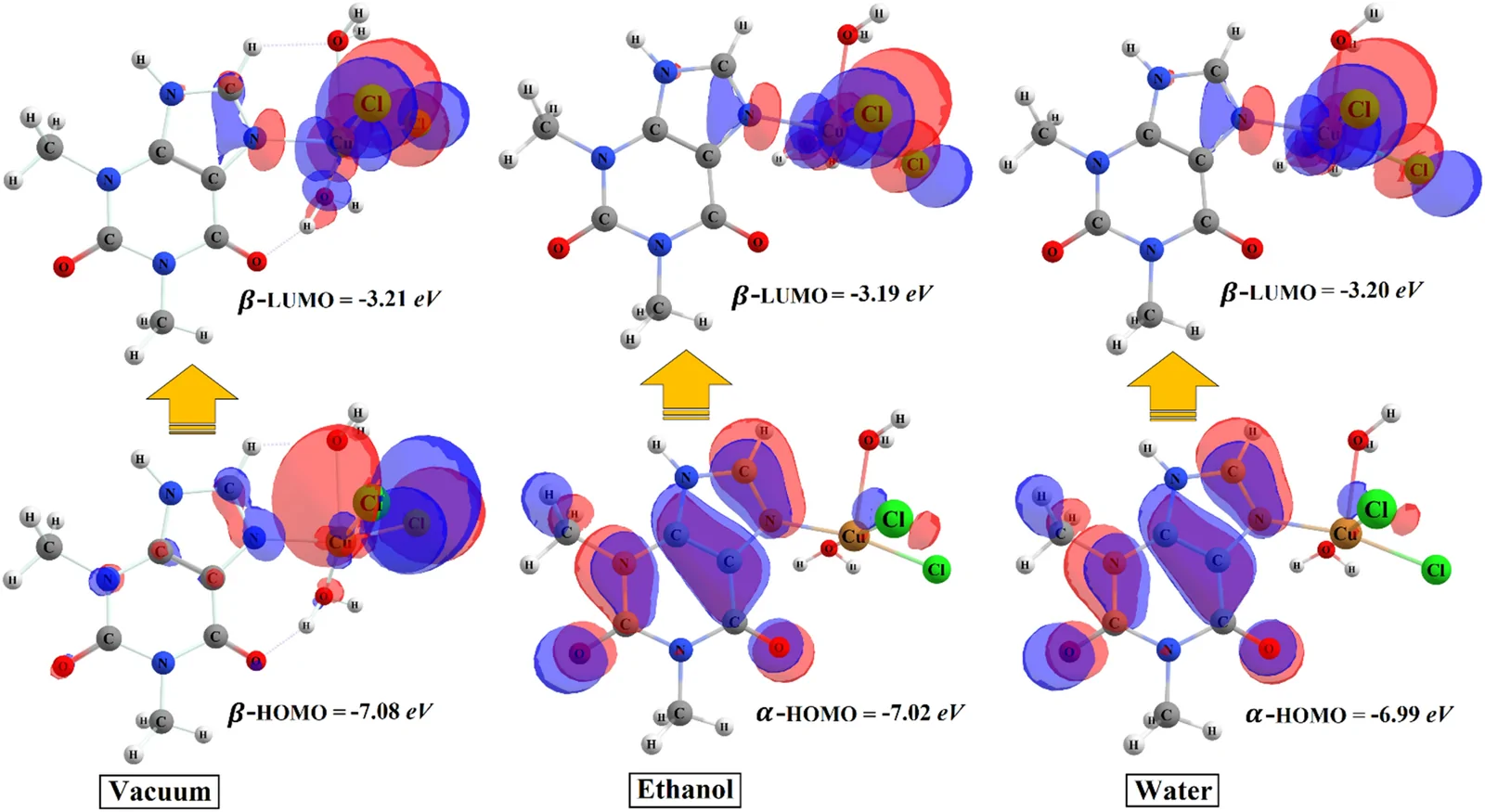
Isosurfaces of frontier molecular orbitals (HOMO and LUMO) for the [Cu(theo)(H_2_O)_2_Cl_2_] complex calculated in vacuum, ethanol and water.

Fig. 5 displays the HOMO and LUMO isosurfaces of the [Cu(theo)(H_2_O)_2_Cl_2_] complex calculated in vacuum, ethanol, and water environments. The *α*-HOMO isosurface is predominantly localized on the theo ligand when calculated in ethanol and water, with energy values of −7.02 eV and −6.99 eV, respectively. In contrast, the *β*-HOMO is localized around the metal center of the complex under vacuum conditions, with an energy of −7.08 eV. Similarly, the *β*-LUMO isosurface is also localized around the metal center, with energy values of −3.21 eV, −3.19 eV, and −3.20 eV for vacuum, ethanol, and water, respectively. The energy values of the frontier molecular orbitals (HOMO and LUMO) are key parameters for understanding intermolecular interactions, as they are directly related to electron donor and acceptor properties.^46^

Reactivity descriptors are essential for understanding the behavior of complexes in biological systems. The system must exhibit adequate reactivity while maintaining sufficient stability to preserve its structural integrity.^47^ The HLG values calculated in ethanol, water, and vacuum were 3.83 eV, 3.79 eV, and 3.87 eV, respectively. These values indicate good electronic stability, consistent with previous studies on copper-based coordination compounds.^48^ The IP and EA represent the energy required to remove an electron from the complex and the energy change upon electron acceptance, respectively.^49^ The values under vacuum and solvation conditions were similar, with differences of less than 0.10 eV. The *χ* and *µ* parameters of the complex were 5.10 eV and −5.10 eV in ethanol and water and 5.15 eV and −5.15 eV in vacuum. The positive electronegativity values indicate the compound's tendency to attract electrons, whereas the negative chemical potential indicates its stability and tendency to retain electrons.^50^ Additionally, the *ω* descriptor is a key parameter for predicting biological activity, as it quantifies the tendency of a system to accept electron density and the associated stabilization energy.^51^ The *ω* values were 6.80 eV in ethanol and 6.84 eV in water and vacuum, which are comparable to or higher than those reported for other theo-containing coordination complexes.^18,52^ The HOMO–LUMO gap and electrophilicity index obtained here are of the same order as those reported for related DFT-characterized heterocyclic systems,^53–55^ confirming a comparable kinetic stability, while the somewhat higher electrophilicity is consistent with the electron-accepting character of the copper(ii) center.

**Fig. 6:**
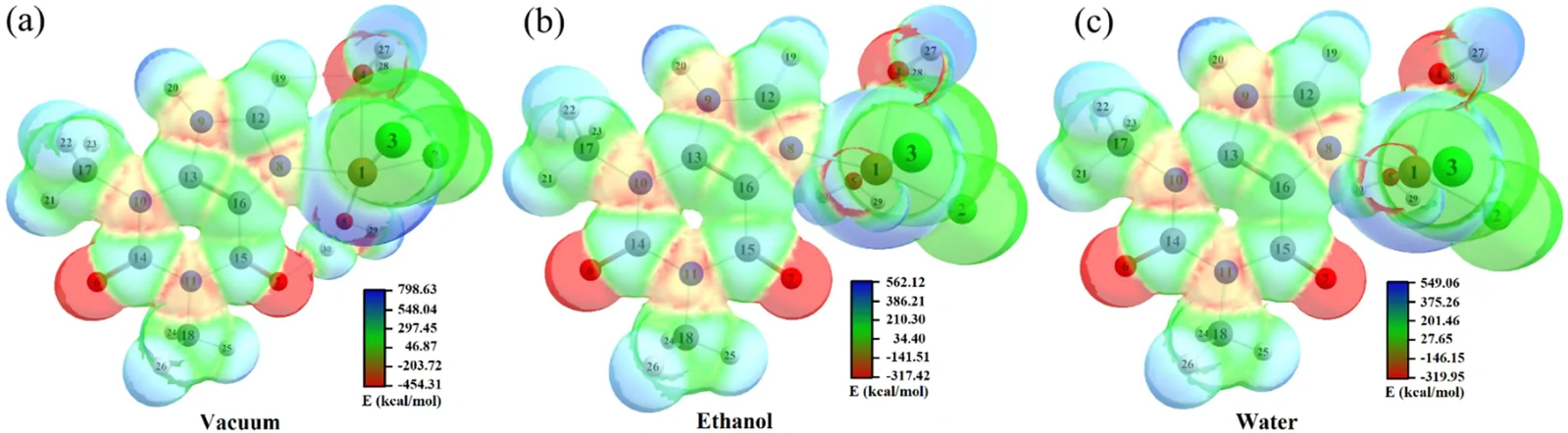
MEP maps of the [Cu(theo)(H_2_O)_2_Cl_2_] complex calculated in (a) vacuum, (b) ethanol, and (c) water environments. The color scale ranges from blue (positive potential values) to red (negative potential, values).

The MEP map was calculated to analyze the charge distribution in the [Cu(theo)(H_2_O)_2_Cl_2_] complex. Fig. 6 displays the MEP maps, in which cold colors (blue and green) indicate positive or neutral regions, whereas hot colors (red and orange) indicate more negative regions. The regions involving oxygen atoms (in the carbonyl groups and coordinated H_2_O molecules) exhibit higher electron density, indicating nucleophilic character. Notably, the MEP maps in water and ethanol show similar potential distributions, with small energy differences, whereas the MEP in vacuum exhibits greater energy variation than in the solvated systems. This electron density mapping allows for the identification of potential sites for intermolecular interactions within the coordination complex, as well as its interactions with biological macromolecules. Consequently, it provides insight into how the complex may interact with molecular targets in biological systems through electrostatic and hydrogen bonding interactions.

### 3.4 Group theory, FT-IR and Raman spectroscopy studies

The XRPD and Rietveld refinement results revealed that the [Cu(theo)(H_2_O)_2_Cl_2_] complex consists of 30 atoms, crystallizing in the triclinic crystal system with space group *P*1̄ (*C*^1^_*i*_). The unit cell contains two formula units (*Z* = 2), totaling 60 atoms. Consequently, based on group theory, the crystal has 180 vibrational degrees of freedom. The decomposition into irreducible representations was obtained by the nuclear site-group (correlation) method.^56^ In the *P*1̄ (*C*^1^_*i*_) structure with *Z* = 2, the asymmetric unit comprises one formula unit (30 atoms), and all atoms occupy the general Wyckoff position 2i (site symmetry *C*_1_). The special positions corresponding to the inversion centres (Wyckoff 1a–1h, site symmetry *C*_i_) are unoccupied. For an atom on a *C*_1_ site, the three translational degrees of freedom span the totally symmetric species A of *C*_1_, which correlates with the factor group *C*_*i*_ as A(*C*_i_) → A_g_ + A_u_. Each independent atom therefore contributes 3A_g_ + 3A_u_, and summing over the 30 atoms of the asymmetric unit gives *Γ*^total^ = 30 × (3A_g_ + 3A_u_) = 90A_g_ + 90A_u_. The balanced gerade (g)/ungerade (u) distribution is a direct consequence of all atoms lying on general positions, that is, each atom belongs to an inversion-related pair, so that in-phase and out-of-phase combinations occur in equal numbers. An atom located on an inversion center would contribute only A_u_ and would break this balance. Since the three acoustic translations transform as A_u_ in *C*_i_, *Γ*^acoustic^ = 3A_u_ and *Γ*^optical^ = 90A_g_ + 87A_u_. Owing to the centrosymmetric space group, the A_g_ modes are Raman-active and the A_u_ modes IR-active (mutual exclusion rule).

**Fig. 7:**
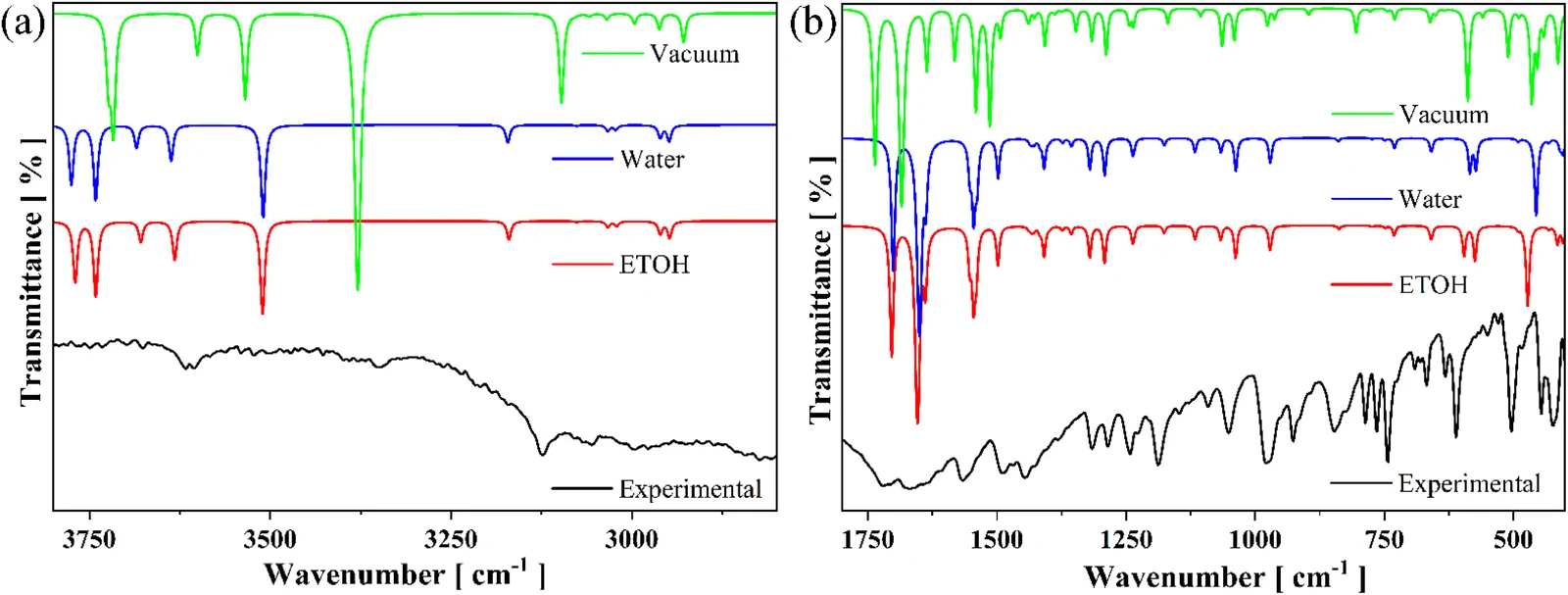
Experimental and DFT-calculated FT-IR spectra in the spectral region of (a) 3800–2850 cm^−^^1^ and (b) 1800–400 cm^−^^1^ of the [Cu(theo)(H_2_O)_2_Cl_2_] complex in vacuum, ethanol, and water environments.

FT-IR spectroscopy was performed to identify and characterize the functional groups present in the [Cu(theo)(H_2_O)_2_Cl_2_] complex. Fig. 7 displays the experimental and DFT-calculated FT-IR spectra in ethanol and water solvation environments, as well as under vacuum conditions, covering the spectral range from 4000 to 400 cm^−1^. The observed vibrational bands and their assignments are summarized in Table 5.

**Table 5:** Assignment of the vibrational modes of the [Cu(theo)(H_2_O)_2_Cl_2_] complex observed in FT-IR and Raman spectra^a^

*ω*_Raman_ [cm^−1^]	*ω*_FT-IR_ [cm^−1^]	*ω*_E__thanol_ [cm^−1^]	*ω*_Water_ [cm^−1^]	*ω*_Vacuum_ [cm^−1^]	Vibrational modes		
					Ethanol	Water	Vacuum
		3929	3935	3881	*v*_a_ (H27O4H28) [94]	*v*_a_ (H27O4H28) [92]	*v*_a_ (H29O4H30) [85]
		3900	3900	3874	*v*_a_ (H29O5H30) [81]	*v*_a_ (H29O5H30) [87]	*v*_a_ (H27O4H28) [77]
	3617	3835	3841	3752	*v*_s_ (H27O4H28) [75]	*v*_s_ (H27O4H28) [93]	*v*_s_ (H27O4H28) [94]
	3605	3786	3791	3684	*v*_s_ (H29O5H30) [92]	*v*_s_ (H29O5H30) [73]	*v* (N9H20) [96]
3529	3349	3659	3658	3522	*v* (N9H20) [90]	*v* (N9H20) [89]	*v*_a_ (H29O4H30) [55]
3157	3123	3304	3306	3228	*v* (C12H19) [19]	*v* (C12H19) [79]	*v* (C12H19) [55] + *γ* (H20N9C12H19) [16] + *γ*_out_ (N8C12N9H19) [16]
		3207	3207	3210	*v* (C18H24) [16] + *v* (C18H25) [53] + *v* (C18H26) [18]	*v* (C18H24) [16] + *v* (C18H25) [54] + *v* (C18H26] [17]	*v* (C18H24) [17] + *v* (C18H25) [61] + *v* (C18H26] [22]
3086		3206	3206	3188	*v* (C17H21) [61] + *v* (C17H22) [17] + *v* (C18H23) [16]	*v* (C17H21) [59] + *v* (C17H22) [15] + *v* (C17H23) [16]	*v* (C17H21) [67] + *v* (C17H22) [16] + *v* (C17H23) [15]
3044	3055	3162	3162	3163	*v*_a_ (H24C18H26) [94]	*v*_a_ (H24C18H26) [94]	*v*_a_ (H24C18H26) [90]
3010	2995	3149	3150	3123	*v*_a_ (H22C17H23) [94]	*v*_a_ (H22C17H23) [90]	*v*_a_ (H22C17H23) [95]
2962		3086	3086	3087	*v* (C18H24) [38] + *v* (C18H25) [25] + *v* (C18H26) [37]	*v* (C18H24) [38] + *v* (C18H25) [24] + *v* (C18H26) [37]	*v* (C18H24) [37] + *v* (C18H25) [23] + *v* (C18H26) [32]
	2824	3072	3073	3052	*v* (C17H21) [39] + *v* (C17H22) [37] + *v* (C17H23) [21]	*v* (C17H21) [21] + *v* (C17H22) [38] + *v* (C17H23) [39]	*v* (C17H21) [20] + *v* (C17H22) [39] + *v* (C17H23) [40]
1706	1722	1776	1773	1810	*v* (O6C14) [30] + *v* (O7C15) [30]	*v* (O6C14) [29] + *v* (O7C15) [28] + *v* (C15C16) [9]	*v* (O6C14) [46] + *v* (O7C15) [17]
1664	1669	1724	1720	1756	*v* (O6C14) [30] + *v* [O7C15] [25]	*v* (O6C14) [30] + *v* (O7C15) [25] + *v* (C15C16) [9]	*v* (O6C14) [16] + *v* (O7C15) [34] + *v* (C15C16) [11]
		1708	1707	1705	*v* (N10C13) [20] + *v* (C13C16) [14]	*v* (N10C13) [19] + *v* (C13C16) [12]	*v* (N10C13) [19] + *v* (C13C16) [15] + *v* (O7C15) [10] + *δ* (N9C13C16) [9]
1611		1618	1618	1649	sc (H27O4H28) [42]	sc (H27O4H28) [33]	sc (H27O4H28) [100]
1569	1567	1611	1610	1606	*v* (N8C12) [12] + *v* (O7C15) [11] + *v* (N9C13) [9] + *δ* (N8C16C13) [9]	*v* (O7C15) [11] + *δ* (C16N8C12) [9] + *v* (C13C16) [9] +*δ* (C12N8C16) [9]	*v* (C13C16) [9] + *v* (O7C15) [9]
		1605	1604	1578	sc (H29O5H30) [33]	sc (H29O5H30) [30]	sc (H29O5H30) [20] + *v* (O7C15) [11]
	1489	1561	1561	1556	*v* (N8C12) [25]	*v* (N8C12) [23]	*v* (N8C12) [23] + *v* (C13C16) [10]
		1499	1497	1503	*τ* (H21C17H23) [16]	wag (H21C17H23) [17] + *τ* (H22C17H23) [9]	sc (H24C18H26) [21] + *δ* (N11C18H25) [9]
1484	1466	1493	1493	1500	sc (H24C18H26) [18]	sc (H24C18H26) [18]	wag (H21C17H23) [28] + *δ* (N10C17H23) [15] + *τ* (H22C17H23) [13]
		1489	1488	1499	sc (H21C17H22) [18] + *τ* (H22C17H23) [11] + *δ* (N10C17H23) [10]	sc (H21C17H22) [17] + *δ* (N10C17H23) [10] + *τ* (H22C17H23) [10]	sc (H22C17H23) [17]
1448		1477	1476	1488	sc (H24C18H25) [17] + wag (H25C18H26) [17]	wag (H25C18H26) [30] + sc (H24C18H25) [29] + *δ* (N11C18H25) [12] + *δ* (N8C18H26) [11]	*τ* (H24C18H26) [16] + *δ* (N11C18H24) [13] + sc (H24C18H25) [11]
	1444	1468	1468	1467	*v* (N8C16) [10]	*v* (N8C16) [10] + *v* (C13C16) [9]	*v* (N9C13) [9] + *v* (N8C16) [9]
1424		1458	1458	1446	*v* (N9C12) [11] + *δ* (N9C12H20) [11] + *v* (N8C16) [10]	*v* (N9C12) [11] + *δ* (N9C12H20) [11]	*v* (N9C12) [11] + *δ* (C13N9H20) [10] + *δ* (N8C12N9) [9] + wag (H21C17H23) [9] + wag (H21C17H22) [9]
1381		1432	1431	1433	*v* (N8C16) [9] + *δ* (N11C18H25] [9]	*δ* (N11C18H25) [10]	*v* (N8C16) [9] + *δ* (N11C18H25) [9]
1358		1413	1413	1404	*δ* (theo_ring_) [49]	*δ* (theo_ring_) [48]	*δ* (theo_ring_) [36]
1297	1315	1376	1376	1372	*δ* (theo_ring_) [54]	*δ* (theo_ring_) [49]	*δ* (theo_ring_) [47]
1287	1285	1346	1346	1343	*δ* (theo_ring_) [61]	*v* (N9C13) [10] + *v* (C13C16) [9]	*δ* (theo_ring_) [65]
		1290	1289	1297	*v* (N11C14) [14] + *v* (N11C15) [9]	*v* (N11C14) [14] + *v* (N11C15) [10]	*v* (N11C15) [10] + *v* (N11C14) [10]
1249	1242	1287	1287	1288	*δ* (theo_ring_) [49]	*δ* (theo_ring_) [49]	*δ* (theo_ring_) [45]
1189	1148	1226	1226	1218	*v* (N9C13) [14] + *δ* (C13N9H20) [10]	*v* (N9C13) [14]	*v* (N9C13) [12]
1128		1164	1164	1153	*v* (N9C12) [21] + *v* (N8C16) [9]	*v* (N9C12) [20]	*δ* (N11C18H24) [13] + *δ* (N11C18H26) [11]
		1149	1149	1152	*δ* (N11C18H26) [17] + *δ* (N11C18H24) [16] + *γ* (O7C15N11C8) [10]	*δ* (N11C18H24) [16] + *δ* (N11C17H26) [15]	*v* (N9C12) [17] + *δ* (C12N8C16) [10]
		1146	1146	1149	*δ* (N10C17H23) [17] + *δ* (N10C17H22) [16] + *γ* (O6C14N10C17) [10] + *δ* (N11C18H24) [9] + *δ* (N11C18H22) [9]	*δ* (N10C17H23) [19] + *δ* (N10C17H22) [18] + *γ* (O6C14N10C17) [10]	*δ* (N10C17H22) [10] + *δ* (N10C17H23) [10] + *v* (N9C12) [9]
1085	1092	1112	1111	1109	*δ* (theo_ring_) [36]	*δ* (theo_ring_) [54]	*δ* (theo_ring_) [58]
1052	1055	1082	1081	1084	*δ* (theo_ring_) [43]	*δ* (theo_ring_) [43]	*δ* (theo_ring_) [42]
	979	1012	1012	1017	*δ* (theo_ring_) [36]	*δ* (theo_ring_) [35]	*v* (N10C17) [12] + *v* (N11C18) [11]
969		1010	1010	1003	*δ* (theo_ring_) [53]	*δ* (theo_ring_) [59]	*δ* (theo_ring_) [40]
928	926	951	951	949	*δ* (theo_ring_) [58]	*δ* (theo_ring_) [49]	*δ* (C12N9C13) [9]
	846	873	875	934	*γ* (C12N8C16C13) [29] + *γ*_out_ (N9C13N10C16) [15] + *γ* (C12N9C13N10) [14] + *γ*_out_ (N8C12N9H19) [14] + *γ* (N8C12N9C13) [11] + *γ*_out_ (C13N10C14C17) [9]	*γ* (C12N8C16C13) [25] + *γ*_out_ (N8C12N9H19) [20] + *γ* (N8C12N9C13) [16] + *γ* (C13N9C12H19) [13] + *γ*_out_ (Cu1N8C12C16) [9]	*γ* (C12N8C16C13) [18] + *δ* (N8C12N9) [10]
785	787	806	806	839	*δ* (theo_ring_) [43]	*δ* (theo_ring_) [49]	*δ* (Cu1O5H30) [19]
764	763	780	780	810	*γ*_out_ (N8C16C13C15) [27] + *γ* (N10C14N11C15) [13] + *γ* (C14N10C13C16) [9]	*γ*_out_ (N8C16C13C15) [21] + *γ* (Cu1N8C16C15) [17] + *γ* (Cu1N8C16C13) [15] + *γ* (O7C15N11C14) [11] + *γ* (O6C14N11C15) [9]	*δ* (O7C15N11) [9]
	742	762	761	776	*γ* (O7C15N11C14) [18] + *γ*_out_ (O7C15N11C16) [13] + *γ*_out_ (C14N11C15C18) [12] + *γ* (O6C14N10C13) [11] + *γ*_out_ (C13N10C14C17) [10]	*γ* (O7C15N11C14) [26] + *γ*_out_ (O7C15N11C14) [19] + *γ* (N11C15C16C13) [15] + *γ* (O6C14N10C13) [14]	*γ*_out_ (N8C16C13C15) [21] + *γ* (N10C14N11C15) [21] + *γ*_out_ (C13N10C14C17) [15] + *γ*_out_ (O7C15N11C16) [10]
691	691	688	688	761	*γ*_out_ (N9C13N10C10) [10] + *γ* (O7C15C16N8) [8]	*γ*_out_ (N9C13N10C16) [10] + *γ* (N10C14N11C15) [9]	*γ* (C13N10C17H21) [15] + *γ* (O6C14N10C13) [15] + *γ* (C14N10C17H21) [15] + *γ* (O7C15N11C14) [12] + *γ*_out_ (C14N11C15C18) [11] + *γ*_out_ (C14N10C14C17) [10] + *γ*_out_ (O7C15N11C16) [9]
667	667	685	686	689	*δ* (theo_ring_) [25]	*δ* (theo_ring_) [41]	*δ* (theo_ring_) [33]
635	632	666	665	678	*γ* (N8C12N9C13) [18] + *γ* (C12N8C16C13) [13] + *γ* (N10C14N11C15) [8]	*γ*_out_ (Cu1N8C12C16) [26] + *γ* (C12N8C16C15) [15]	*γ*_out_ (N9C13N10C16) [9]
610	612	621	609	660	*δ* (Cu1O5H29) [15] + *δ* (Cu1O5H30) [13]	*δ* (Cu1O5H29) [14] + *δ* (Cu1O5H30) [13]	*γ* (C12N8C16C15) [10] + *γ* (N8C12N9C13) [9]
571	549	599	597	613	*γ* (C16N8C12N9) [14] + *γ* (N8C12N9C13) [11] + *γ* (C12N8C16C15) [9] + *γ* (H20N9C13C16) [9] + *γ* (N9C13C16C15) [8]	*γ* (N9C13N10C17) [18] + *γ*_out_ (Cu1N8C12C16) [11]	*δ* (Cu1O5H29) [12]
554	528	580	579	583	*br* (theo_ring_) [71]	*br* (theo_ring_) [73]	*br* (theo_ring_) [49]
503	503	511	511	531	*br* (theo_ring_) [75]	*br* (theo_ring_) [71]	*v* (Cu1N8) [27] + *δ* (Cl3Cu1O5) [23] + *v* (Cu1O5) [23]
492	445	492	475	510	*δ* (Cu1O5H30) [13] + *δ* (Cu1O5H29) [10]	*δ* (Cu1O5H30) [13] + *δ* (Cu1O5H29) [10] + *γ* (C16N8C12H19) [9]	*γ* (H20N9C13C16) [16] + *γ* (N8C16C13N9) [15] + *γ* (N8C12N9C13) [15] + *γ* (N9C13N10C17) [10] + *γ* (C17N10C13C16) [10]
446	422	450	450	484	*br* (theo_ring_) [62]	*br* (theo_ring_) [60]	*br* (theo_ring_) [48]
		432	427	473	*δ* (Cu1O4H27) [17] + *δ* (Cl2Cu1O4) [15] + *δ* (Cu1O4H28) [9] + *γ* (Cu1N8C16C13) [8] + *γ* (C12N8C16C13) [8]	*δ* (Cu1O4H28) [26] + *δ* (Cu1O4H27) [24]	*γ* (O5Cu1N8C12) [11] + *γ* (C12N8C16C13) [10]
363		421	421	460	*δ* (theo_ring_) [60]	*δ* (theo_ring_) [76]	*δ* (theo_ring_) [57]
		383	383	431	*δ* (O7C15N11) [20] + *δ* (C15N11C18) [15]	*δ* (theo_ring_) [36]	*δ* (theo_ring_) [62]
		365	363	426	*δ* (Cu1O4H28) [15] + *δ* (Cl2Cu1O4) [8]	*γ* (C12N9C13N10) [10]	*δ* (O6C14N11) [14] + *δ* (C14N11C15) [14]
		363	344	404	*γ*_out_ (C13N10C14C17) [13] + *γ* (N8C16C13N9) [11] + *γ* (C14N11C15C16) [8]	*δ* (Cu1O4H27) + *δ* (Cu1O4H28)	*δ* (theo_ring_) [58]
		341	341	370	*δ* (theo_ring_) [42]	*δ* (theo_ring_) [47]	*v* (Cu1Cl2) [20] + *v* (Cu1O5) [17] + *v* (Cu1Cl3) [15]
302		323	320	355	*v*_a_ (Cl2Cu1Cl3) [37] + *v* (Cu1O5) [24]	*v* (Cu1O5) [25] + *v* (Cu1Cl2) [25] + *v* (Cu1Cl3) [14]	*γ* (C13N10C14C17) [15] + *γ* (N8C16C13N9) [14] + *v* (Cu1 Cl3) [10] + *γ* (C12N9C13N10) [9] + *γ* (C14N10C13C16) [9]
		311	309	347	*δ* (theo_ring_) [34]	*δ* (theo_ring_) [55]	*v* (Cu1Cl2) [12] + *v* (Cu1Cl3) [11]
		299	297	340	*v* (Cu1Cl2) [19] + *v* (Cu1Cl3) [19] *δ* (C13N10C17) [10] + *δ* (N9C13N10) [9]	*v* (Cu1Cl2) [19] + *v* (Cu1Cl3) [18] + *δ* (C13N10C17) [11] + *δ* (N9C13N10) [10]	*v* (Cu1Cl2) [12] + *δ* (C14N11C18) [10] + *δ* (N11C15C16) [10]
		279	280	313	*γ*_out_ (C14N11C15C18) [19] + *γ* (N10C14N11C15) [12] + *γ* (C13N10C14N11) [10] + *γ* (C18N11C15C16) [10] + *γ* (C14N10C13C16) [8]	*γ*_out_ (C14N11C15C18) [18] + *γ* (C13N10C14N11) [13] + *γ* (N10C14N11C18) [10]	*γ*_out_ (C13N10C14C17) [15] + *v* (Cu1Cl3) [12] + *τ* (H27O4H28) [11]
		251	250	297	*v* (Cu1Cl3) [13] + *v* (Cu1O5) [10] + *v* (Cu1N8) [10] + *δ* (N8C16C13) [10]	*v* (Cu1Cl3) [14] + *v* (Cu1O5) [10] + *v* (Cu1N8) [10]	*δ* (theo_ring_) [29]
211		244	238	285	*γ* (O5Cu1O4H27) [10] + *γ* (N8Cu1O4H27) [9]	*δ* (O5Cu1N8) [17] + *γ* (N10C13C16C15) [12] + *δ* (Cl3Cu1N8) [10]	*v* (Cu1Cl3) [19] + *v* (Cu1O5) [15]
		229	218	265	*γ*_out_ (C13N10C14C17) [9] + *v* (Cu1Cl3) [8] + *δ* (Cl3Cu1N8) [8]	*δ* (Cl2Cu1O5) [16]	*γ*_out_ (C14N11C15C18) [18] + *γ* (C13N10C14N11) [12] + *γ* (C14N11C15C16) [12] + *γ* (C12N8C16C15) [10]
				236	Lattice modes	Lattice modes	*δ* (Cl3Cu1N8) [10] + *γ* (Cu1N8C12N9) [10]
				221	Lattice modes	Lattice modes	*γ* (C12N8C16C13) [9]
169					Lattice modes	Lattice modes	Lattice modes
136					Lattice modes	Lattice modes	Lattice modes
127					Lattice modes	Lattice modes	Lattice modes
116					Lattice modes	Lattice modes	Lattice modes
103					Lattice modes	Lattice modes	Lattice modes
92					Lattice modes	Lattice modes	Lattice modes
86					Lattice modes	Lattice modes	Lattice modes
72					Lattice modes	Lattice modes	Lattice modes
64					Lattice modes	Lattice modes	Lattice modes
49					Lattice modes	Lattice modes	Lattice modes
37					Lattice modes	Lattice modes	Lattice modes

a *v*_s_: symmetrical stretching; *v*_as_: antisymmetric stretching; *r*: rocking; sc: scissoring; *γ*: torsion; *δ:* angular deformation; *br*: breathing; *γ*_out_: torsion out of plane; *τ*: twistting; wag: wagging.

The spectral region from 4000 to 2800 cm^−1^ corresponds to the stretching vibrations of coordinated H_2_O molecules, amine (N–H), and methyl (C–H) groups. The bands at 3617 and 3605 cm^−1^ are attributed to the antisymmetric and symmetric stretching vibrations of H_2_O molecules coordinated to the copper(II) center. The N–H stretching mode from the theo ligand is observed at 3449 cm^−1^, while C–H stretching vibrations appear at 3123 cm^−1^. Although H_2_O vibrational modes are active in this region, the strong absorption of these molecules leads to significant band overlap. Therefore, complementary spectroscopic techniques, such as Raman spectroscopy, are necessary to fully characterize the vibrational modes associated with the functional groups present in the complex.

In the 1722–1669 cm^−1^ region, the predominant vibrational bands correspond to the carbonyl (C=O) stretching modes, attributed to *ν*(C14=O6) and *ν*(C15=O7). The occurrence of carbonyl stretching vibrations in this spectral region has been reported for other theo-containing complexes.^17,57^ In the calculation performed under vacuum conditions, a band at 1810 cm^−1^ is observed, which results from intramolecular hydrogen bonding interactions involving H30⋯O7, as shown in Fig. 4. Thus, under vacuum conditions, one of the bands corresponding to this functional group is blue-shifted to a higher wavenumber due to the formation of this hydrogen bond.

In the 1567–926 cm^−1^ region, most bands are attributed to C–N and C–C stretching vibrations within the theo ring system, specifically *ν*(C–N). Additionally, vibrational modes corresponding to in-plane bending deformations *δ*(N–C–H) and *δ*(H–C–H) from the theo ligand are observed.

In the 846–422 cm^−1^ region, angular deformations, in-plane twisting, and out-of-plane bending modes are predominant. Out-of-plane wagging vibrations are observed at 763, 742, and 691 cm^−1^. Notably, in this region, metal–ligand stretching vibrations *ν*(Cu–N) and *ν*(Cu–O) appear as weak bands at 612 and 445 cm^−1^, respectively, which are characteristic of metal coordination. For aromatic compounds, ring breathing modes are expected in this spectral region; for the theo ligand, these are observed at 528, 503, and 422 cm^−1^, corresponding to breathing vibrations of the imidazole and pyrimidine rings.

The calculations performed in ethanol and water solvation environments show good agreement with the experimental results. However, the calculation performed under vacuum conditions exhibits noticeable band shifts. This effect is particularly evident in the 4000–2800 cm^−1^ region, where O–H and N–H stretching modes are red-shifted to lower wavenumbers, and in the carbonyl stretching mode, which is blue-shifted to higher wavenumbers (1810 cm^−1^). These findings demonstrate that solvation effects significantly influence the calculated vibrational frequencies, highlighting the importance of including solvent models to accurately reproduce experimental spectra.

**Fig. 8:**
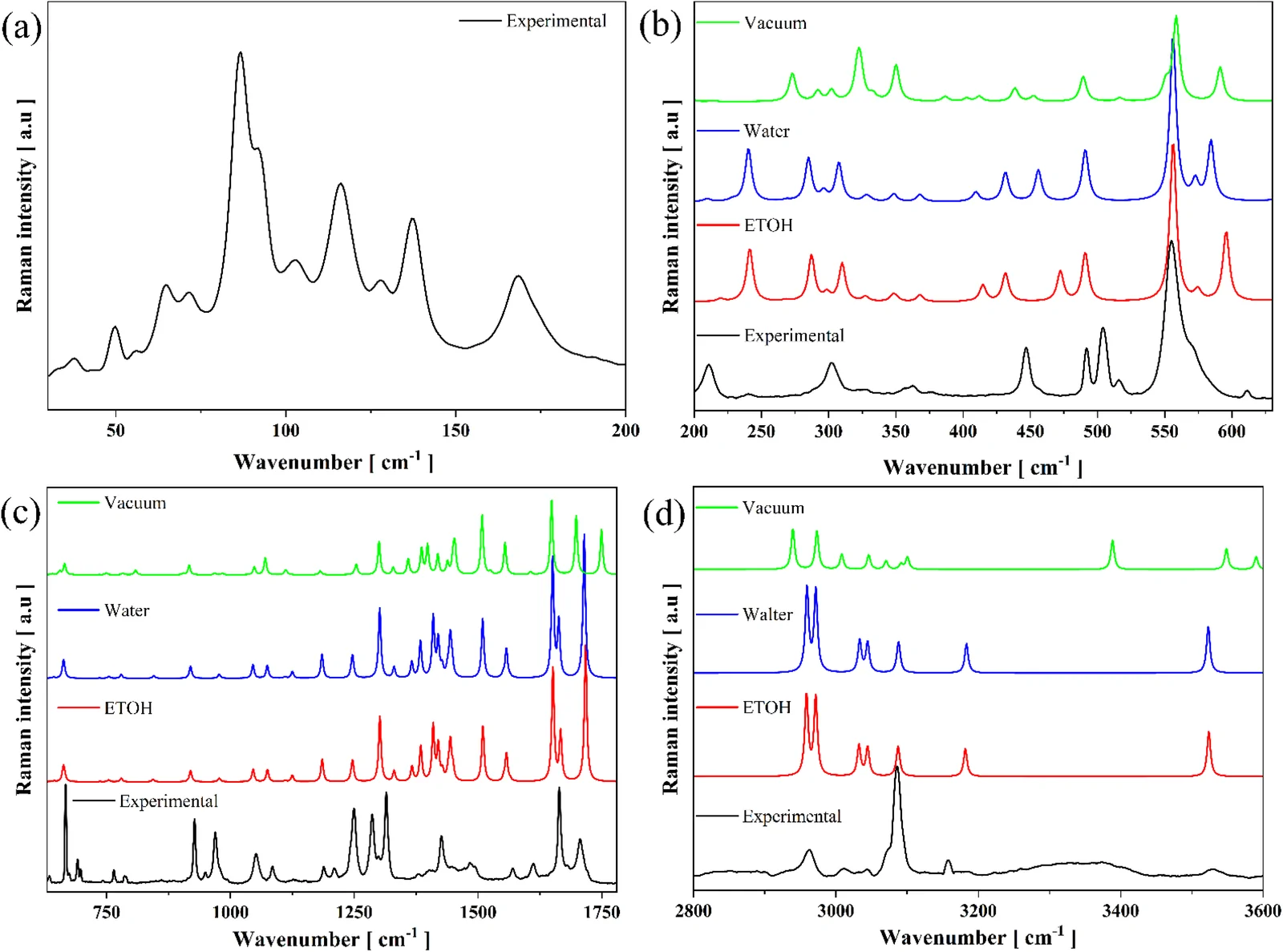
Experimental and DFT-calculated Raman spectra of the [Cu(theo)(H_2_O)_2_Cl_2_] complex in vacuum, ethanol, and water environments: (a) lattice modes (30–200 cm^−1^), (b) 200–630 cm^−1^, (c) 630–1800 cm^−1^, and (d) 2800–3600 cm^−1^.

The experimental and DFT-calculated Raman spectra of the [Cu(theo)(H_2_O)_2_Cl_2_] complex are shown in Fig. 8. The spectral regions analyzed are 30–200 cm^−1^, 200–630 cm^−1^, 630–1800 cm^−1^, and 2800–3600 cm^−1^. The assignments of the vibrational modes are presented in Table 5.

Fig. 8(a) shows the experimental spectrum in the 30–200 cm^−1^ region, a spectral range corresponding to external modes or lattice vibrations. The calculated spectra for the isolated asymmetric unit in this region generally does not match the experimental spectrum, as these modes arise from intermolecular interactions and collective vibrations of the entire crystal lattice. Since the DFT calculations were performed only on the isolated molecular complex, intermolecular and crystal packing effects were not considered. Therefore, the vibrational bands observed below 200 cm^−1^ were assigned exclusively to lattice modes.

Stretching vibrations involving the copper ion and chloride ligands are expected in the 290–500 cm^−1^ region.^58^ In the DFT calculations, Cu–Cl stretching modes are attributed to bands at 251, 299, and 323 cm^−1^. In addition to these metal–halide vibrations, Fig. 8(b) also shows bands resulting from deformations in the coordination sphere of the metal ion, involving Cu–O–H bending modes, which were observed at 610 and 492 cm^−1^.

Vibrations at 446, 503, and 554 cm^−1^ are attributed to ring breathing modes of the theo aromatic rings. Ring breathing vibrations involve symmetric in-phase stretching of the C–C and C–N bonds in heterocyclic ring systems. In the theo cocrystal with monohydrated resorcinol, breathing modes of the theo aromatic rings are observed at 452 and 566 cm^−1^.^59^

The spectral region from 630 to 1710 cm^−1^ of the Raman spectrum is shown in Fig. 8(c). This region, known as the fingerprint region, contains bands that are characteristic of the molecular structure and the measurement environment.^60,61^ The predominant bands arise from C–C and C–N bond stretching and angular deformations of C–H, CH_2_, and CH_3_ groups. Notably, the carbonyl stretching band can be identified from 1654 cm^−1^ onwards.

Angular deformations of C–H, CH_2_, and CH_3_ functional groups were observed between 1400 and 1500 cm^−1^. Scissoring and wagging deformations were assigned to the band at 1448 cm^−1^, while at 1484 cm^−1^, only the scissoring vibration was detected. Additionally, the bending mode of coordinated H_2_O molecules was observed near 1600 cm^−1^, with the band at 1611 cm^−1^ corresponding to the H–O–H scissoring motion.

The vibrational modes experimentally observed at 1706 and 1664 cm^−1^ are attributed to the carbonyl *ν*(C=O) stretching vibrations. The experimental values and those obtained from DFT calculations in solvation environments, after applying the scaling factor mentioned in the methodology, show good agreement. In a vibrational study by Ribeiro-Claro *et al.*,^62^ stretching bands of this functional group were observed at 1665 and 1707 cm^−1^, confirming the consistency of our results with the literature.

The spectral range from 2800 to 3600 cm^−1^ is shown in Fig. 8(d), comparing the experimental and calculated spectra. This region is characterized by stretching vibrations of C–H, N–H, and O–H functional groups. Experimentally, six active bands were identified, exhibiting strong correlation with the calculated spectra when applying the scaling factor.

The bands observed at 2962 and 3086 cm^−1^ exhibit shoulders and are attributed to CH_3_ symmetric and antisymmetric stretching vibrations. Low-intensity bands at 3010 and 3044 cm^−1^ correspond to CH_2_ antisymmetric stretching modes. C–H and N–H stretching vibrations were identified at 3157 and 3529 cm^−1^, respectively.

DFT calculations predict bands corresponding to O–H stretching vibrations of coordinated H_2_O molecules between 3700 and 3900 cm^−1^; however, these bands were not observed experimentally due to potential sample degradation or heating effects from the laser excitation. As a result, the O–H stretching modes of H_2_O molecules are expected to appear at wavenumbers above 3600 cm^−1^, as observed in the FT-IR analysis. The complete assignments of the vibrational modes for the [Cu(theo)(H_2_O)_2_Cl_2_] complex are presented in Table 5.

### 3.5 UV-Vis-NIR spectroscopy

**Fig. 9:**
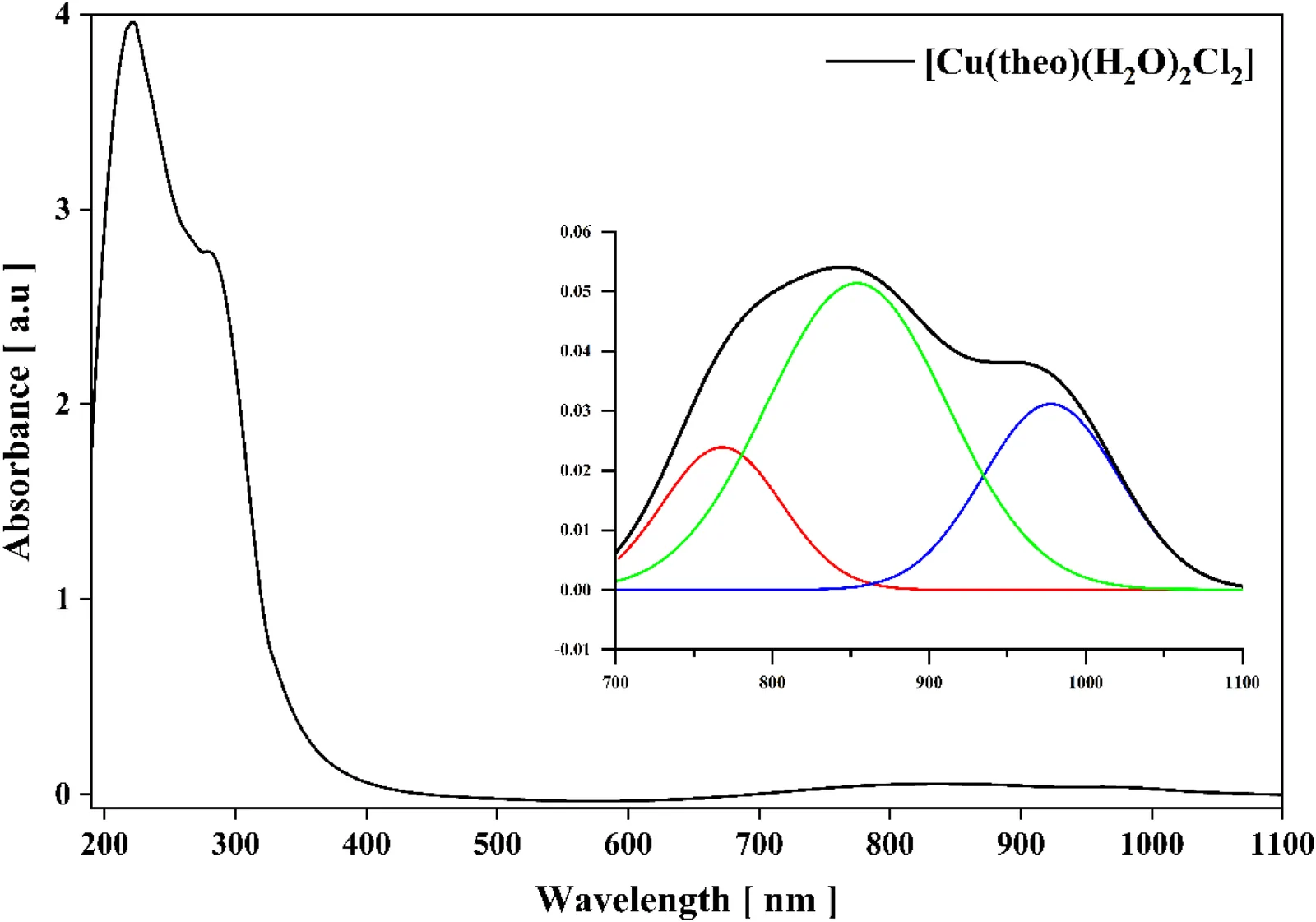
UV-Vis-NIR absorption spectrum of the [Cu(theo)(H_2_O)_2_Cl_2_] complex in the 190–1100 nm range.

The optical absorption spectrum of the [Cu(theo)(H_2_O)_2_Cl_2_] complex is shown in Fig. 9. In the UV region (200–400 nm), two intense bands are attributed to the theo ligand, corresponding to π → π* and *n* → π* transitions, as reported in the literature.^17,52^ In the Vis and NIR regions (400–1100 nm), a broad absorption band is observed, attributed to *d*–*d* electronic transitions of the Cu^2+^ ion. Although *d*–*d* transitions are typically associated with tetracoordinate copper complexes with square-planar or tetrahedral geometries.^18^ The present complex is pentacoordinate with a square-pyramidal geometry but exhibits significant tetrahedral distortion.^19^ The broad band envelope arises from three overlapping electronic transitions: ^2^B_1g_ → ^2^A_1g_, ^2^B_1g_ → ^2^B_2g_, and ^2^B_1g_ → ^2^E_g_, which are spin-allowed but parity-forbidden (Laporte-forbidden).^63^

### 3.6 Molecular docking study

**Fig. 10:**
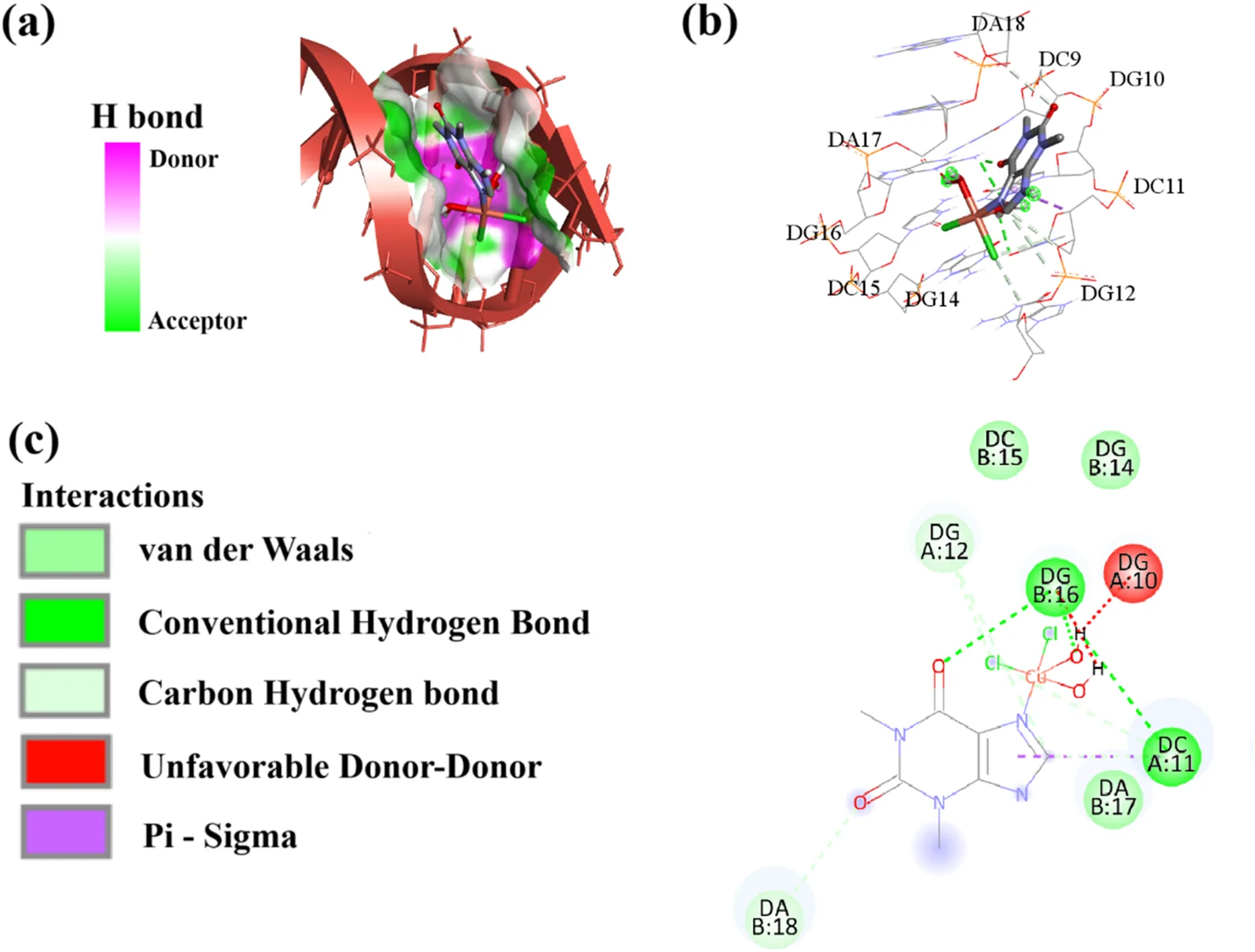
Molecular docking of the [Cu(theo)(H_2_O)_2_Cl_2_] complex with DNA: (a) overall view showing intercalation between base pairs, (b) detailed 3D view of intermolecular contacts, and (c) 2D interaction diagram showing specific contacts with nucleotides.

**Fig. 11:**
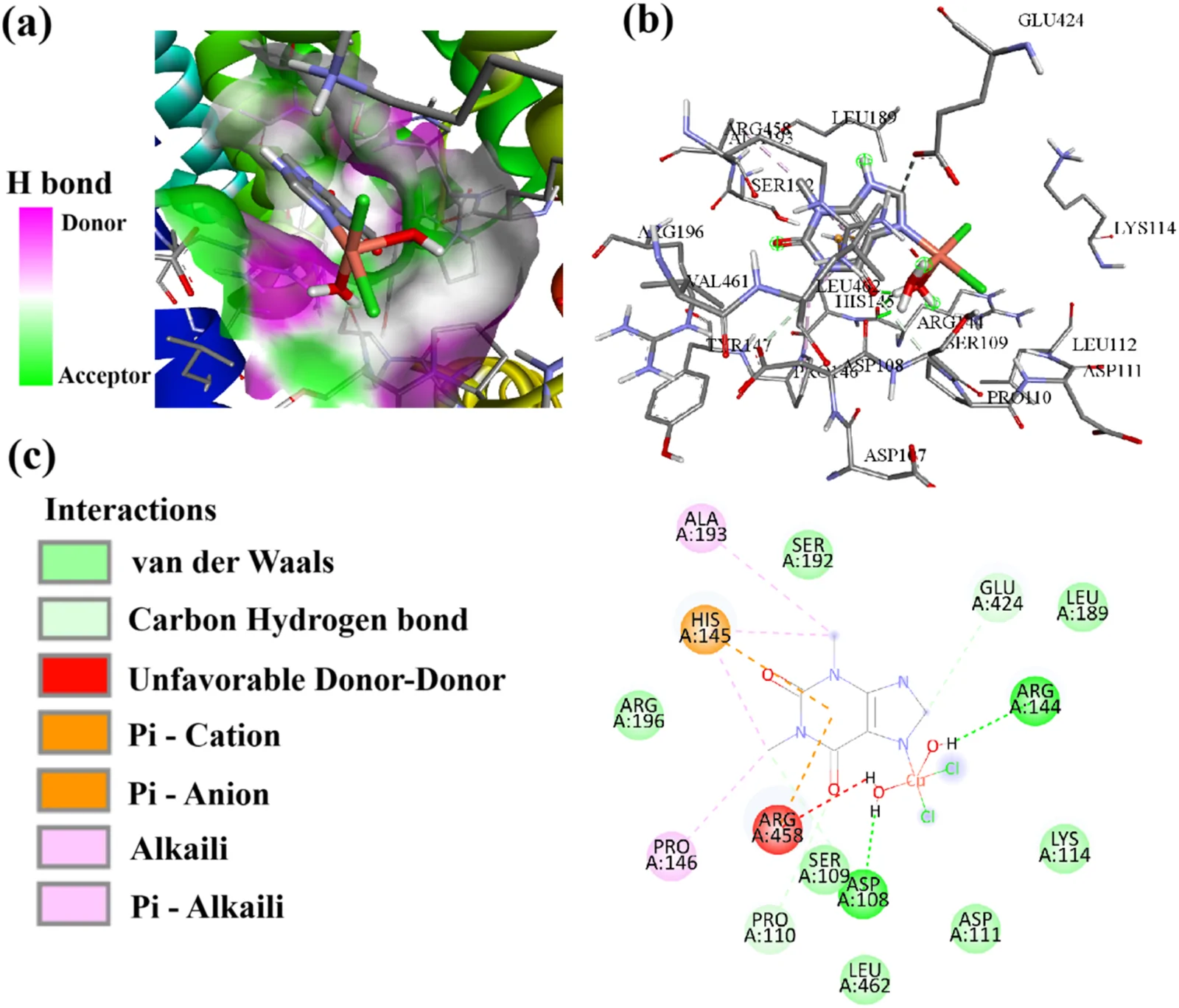
(a) Interactions of the [Cu(theo)(H_2_O)_2_Cl_2_] complex with BSA; (b) details of the intermolecular contacts formed in 3D; (c) 2D intermolecular contacts and their interactions with amino acids, using a grid box spacing of 0.375 Å and grid box dimensions of 126 × 16 × 126 points.

Molecular docking has become a valuable approach for predicting the binding modes and affinities of small molecules and metal complexes toward biological macromolecules and is widely applied in the screening of new anticancer candidates.^64^ Molecular docking studies were conducted to investigate the interactions of the [Cu(theo)(H_2_O)_2_Cl_2_] complex with DNA and BSA in order to identify the preferred binding modes and calculate the binding energies. The interactions of the complex with the DNA dodecamer and BSA are shown in Fig. 10 and 11, respectively. The docking parameters, including *K*_i_, Δ*G*_bind_, estimated intermolecular energy (vdW + H-bond + desolvation), and total intermolecular energy are presented in Table 6.

**Table 6:** Binding parameters for the [Cu(theo)(H_2_O)_2_Cl_2_] complex with DNA and BSA from molecular docking calculations

Macromolecule	Inhibition constant (*K*_i_) [µM]	Binding energy [kcal mol^−1^]	Total energy of vdW + binding + desolvation [kcal mol^−1^]	Total intermolecular energy [kcal mol^−1^]
DNA	5.85	−7.14	−7.75	−7.96
BSA	40.78	−5.99	−6.87	−6.81

The docking results shown in Fig. 10(a) suggest that the lowest-energy pose corresponds to an intercalative binding mode, in which the complex is positioned between adjacent base pairs of the DNA double helix.^65,66^Fig. 10(b) shows an enlarged view of the binding site from the molecular docking calculation, highlighting the nucleotides that interact with the complex. The DNA base pairs interact with the ligand through multiple non-covalent interactions, as shown in the 2D interaction map in Fig. 10(c): van der Waals interactions (DA : B17, DA : B18, DC : B15, DG : B14), hydrogen bonds (DC : A11, DG : B11), carbon–hydrogen bonds (DG : A12), and an unfavorable donor–donor interaction (DA : A10).

Serum albumin is the most abundant plasma protein produced in the liver and plays a crucial role in drug transport and delivery to target sites.^67,68^ Molecular docking calculations with BSA revealed favorable interactions between the complex and this macromolecule, as shown in Fig. 11(a). The amino acid residues in the BSA structure that interact with the complex are displayed in Fig. 11(b), detailing the molecular contacts formed. These interactions occur through van der Waals forces (Asp108, Ser109, Asp111, Leu112, Lys114, Leu189, Arg196, Leu462), hydrogen bonds (Arg144, Ser192), an unfavorable donor–donor interaction (Arg458), and π-cation and π-anion interactions (His145, Glu424), as shown in the 2D interaction diagram in Fig. 11(c).

The molecular docking of the complex with the DNA dodecamer yielded an inhibition constant (*K*_i_) of 5.85 µM and a binding free energy (Δ*G*_bind_) of −7.14 kcal mol^−1^, indicating strong affinity. For BSA, these values were 40.78 µM and −5.99 kcal mol^−1^, respectively, indicating moderate binding affinity. Additional docking parameters are presented in Table 6. Taken together, these *in silico* results suggest that the complex can establish favorable binding interactions with both DNA and BSA, with a predicted preference for DNA, consistent with, but not by itself establishing, potential biological activity.

It should be emphasized that molecular docking provides only a static, *in silico* prediction of the preferred binding geometry and relative affinity toward isolated macromolecular targets. It does not account for solvation dynamics, conformational flexibility, competing cellular targets or cellular uptake, and therefore cannot, on its own, establish the actual mechanism of anticancer action. The present docking data should thus be regarded as supporting evidence for possible DNA- and protein-binding interactions rather than as proof of a defined mode of action. Experimental validation, such as spectroscopic DNA-binding titrations (UV-Vis hypochromic, ethidium bromide displacement and viscosity measurements), protein-binding studies (fluorescence quenching of BSA/HSA) and DNA-cleavage assays, are required to confirm the proposed binding modes.

### 3.7 Cell viability

**Fig. 12:**
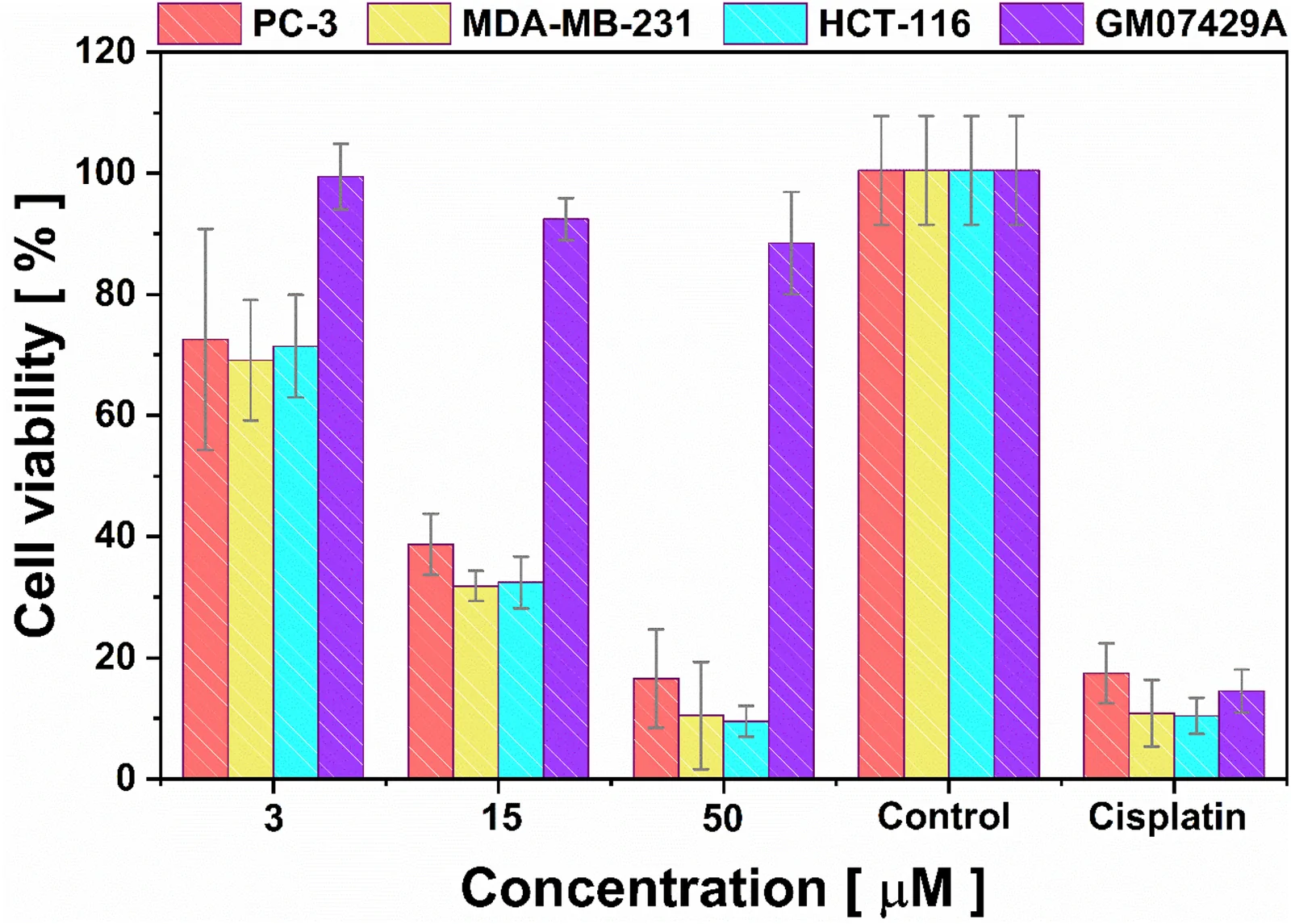
Dose-dependent cytotoxicity of the [Cu(theo)(H_2_O)_2_Cl_2_] complex against tumor cell lines PC-3 (prostate), MDA-MB-231 (breast), HCT-116 (colorectal), and non-tumorigenic cell line GM07429A (lung fibroblast). Cisplatin (5 mM) was used as a positive control. Untreated cells were used as the negative control.

The biological activity of the [Cu(theo)(H_2_O)_2_Cl_2_] complex was evaluated through cytotoxicity assays conducted on three tumor cell lines: PC-3 (prostate), MDA-MB-231 (breast), and HCT-116 (colorectal), as well as on the non-tumorigenic cell line GM07429A (lung fibroblast). The results shown in Fig. 12 demonstrate that the complex inhibits the growth of cancer cell lines in a dose-dependent manner while showing minimal cytotoxicity toward healthy cells. The results were compared with those of cisplatin, a well-established platinum-based antineoplastic agent used against various types of cancer.

The cytotoxic effect of the [Cu(theo)(H_2_O)_2_Cl_2_] complex on cancer cells showed promising results across different concentrations. In all tumor cell lines, the 50 µM concentration exhibited the highest cytotoxic effect. At this concentration, cell viability was reduced to 16.59% for PC-3, 10.49% for MDA-MB-231, and 9.55% for HCT-116, corresponding to approximately 83–90% growth inhibition. Compared to cisplatin, the complex demonstrated comparable cytotoxic activity against tumor cells. However, unlike cisplatin, it showed significantly lower toxicity toward the healthy cell line, suggesting better selectivity for cancer cells.

Following the initial cytotoxicity assessment, the [Cu(theo)(H_2_O)_2_Cl_2_] complex appears to be a promising anticancer candidate, as it showed dose-dependent activity against tumor cells with minimal toxicological risk to normal cells (well above 70% to be considered non-toxic). Based on the experimental data, IC_50_ values were estimated through nonlinear regression analysis, yielding values ranging from 2.91 ± 2.34 to 12.18 ± 1.74 µM. The highest IC_50_ value (12.18 µM ± 1.74) corresponds to the healthy cell line GM07429A, while the lowest value (2.91 µM ± 2.34) was observed for the MDA-MB-231 breast cancer cell line, indicating preferential cytotoxicity toward malignant cells. At the highest tested concentration of 50 µM, the [Cu(theo)(H_2_O)_2_Cl_2_] complex exhibited cytotoxicity on the three cancer cell lines comparable to cisplatin at 5 mM, yet sparing healthy ones.

By comparing the IC_50_ values of tumor cells to those of the non-tumorigenic cell line, the SI was calculated. For all tumor cell lines, the SI values were greater than 1, indicating preferential cytotoxicity toward cancer cells over normal cells. Considering the combined analysis of cell viability, IC_50_ values, and selectivity indices, the cytotoxic activity follows the order: MDA-MB-231 > HCT-116 > PC-3. The IC_50_ and selectivity index values for each cell line are presented in Table 7.

**Table 7:** IC_50_ values and SI for the [Cu(theo)(H_2_O)_2_Cl_2_] complex against tumor cell lines and the non-tumorigenic GM07429A cell line

Cell line	Cell type	[Cu(theo)(H_2_O)_2_Cl_2_] IC_50_ [µM]	SI
PC-3	Prostate	3.77 ± 1.52	3.23
MDA-MB-231	Breast	2.91 ± 2.34	4.19
HCT-116	Colorectal	3.22 ± 3.08	3.78
GM07429A	Lung fibroblast	12.18 ± 1.74	—

*In vitro* cytotoxicity assays using human cell lines represent a crucial initial step in evaluating the anticancer potential of novel compounds, providing essential preliminary data for drug development. The [Cu(theo)(H_2_O)_2_Cl_2_] complex exhibited significantly lower cytotoxicity against healthy cells compared to tumor cells, demonstrating favorable selectivity.

The low-micromolar potency of [Cu(theo)(H_2_O)_2_Cl_2_] (IC_50_ = 2.91 ± 2.34–3.77 ± 1.52 µM) is comparable to, or exceeds, that of previously reported copper(ii) anticancer complexes against the same or related cell lines, as shown in Table 8, and is competitive with cisplatin in these lines.^69–71^ This activity is consistent with a clear structure–activity rationale: the planar aromatic theo (a purine-related methylxanthine) favors DNA intercalation; the labile chloride ligands can aquate to give reactive species, as in cisplatin; and the redox-active Cu^2+^/Cu^+^ couple enables Fenton-like reactive oxygen species (ROS) generation. This behaviour matches the high electrophilicity index (*ω* ≈ 6.8 eV) and moderate HOMO–LUMO gap (3.8 eV) obtained by DFT (Section 3.3.2).

**Table 8:** IC_50_ values and DNA-binding modes of the [Cu(theo)(H_2_O)_2_Cl_2_] complex and representative copper(ii) anticancer complexes reported for the same or related tumor cell lines

Copper(ii) complex	Cell line	IC_50_ [ µM ]	DNA binding/mechanism	Reference
[Cu(theo)(H_2_O)_2_Cl_2_]	MDA-MB-231/HCT-116/PC-3	2.91/3.22/3.77	Intercalation (docking); ROS	This work
[CuL(*o*-phen)]·H_2_O a	MDA-MB-231	2.68	Intercalation	69
Cu^2+^-naphthalimide (series)	MDA-MB-231 (+4 lines)	2.53–19.18	Intercalation; ≈10× cisplatin	70
[Cu(Boc-gly)(phen)_2_]NO_3_^b^	HCT-116	0.6	Intercalation/oxidative cleavage	71
ICA-Cu (dinuclear) c	MDA-MB-231	5.43	Intercalation; •OH/ROS	75

a [Cu(*o*-HO-C_6_H_4_C(H)=N–C_6_H_4_-SH-*o*)(*o*-phenanthroline)]·H_2_O.

b [Cu(Boc-glycine)(2,2-bipyridyl)_2_]NO_3_.

c [Cu_2_(C_9_H_6_O_2_N)_4_(H_2_O)_2_]·2H_2_O.

The selectivity toward malignant cells (SI > 1) reflects the redox vulnerability of tumour cells, which display elevated copper demand (CTR1 up-regulation) and higher basal ROS with weaker antioxidant buffering.^72,73^ Copper species exploit this imbalance, so that the additional oxidative stress preferentially kills cancer cells while sparing normal ones such as the GM07429A fibroblasts. This also accounts for the strongest effect against the triple-negative MDA-MB-231 line (SI = 4.19), reinforced by the 7-fold higher copper accumulation reported for HCT-116 cells relative to normal colon cells.^74^

The electronic descriptors presented in Section 3.3 provide a molecular-level rationale for the activity described here. The MEP map located the electronegative regions on the carbonyl, chloride and aqua oxygen/chlorine atoms (hydrogen-bond acceptors) and the electropositive regions around the copper(ii) center and the coordinated-water hydrogens (hydrogen-bond donors and electrostatic contacts). These reactive sites match the specific hydrogen-bond, van der Waals and electrostatic contacts obtained in the docking with DNA and BSA (Section 3.6). Consistently, the moderate HOMO–LUMO gap (3.8 eV) reflects a species reactive enough to engage biomolecular targets yet stable enough to reach them intact, whereas the high electrophilicity index (6.8 eV), the electronegativity (5.10 eV) and the comparatively soft character (0.26 eV^−1^) denote a pronounced electron-accepting, polarizable species, a profile compatible with intercalative stacking against the DNA π-system and with the redox-accessibility of the Cu^2+^/Cu^+^ couple that can drive Fenton-like ROS generation. These theoretical features thus offer a coherent rationale for the low-micromolar, tumor-selective cytotoxicity reported above, although, as only a single compound was studied, they represent a qualitative interpretation rather than a predictive structure–activity correlation.

A remaining mechanistic question is whether the observed cytotoxicity originates from the intact complex or from Cu^2+^ released through ligand exchange. The present data does not allow these possibilities to be distinguished: both are consistent with the trends discussed above, since the neutral, lipophilic complex could act intact, favoring cellular uptake and theo-mediated intercalation, while partial aquation could release redox-active Cu^2+^*in situ*. It is likely that both contributions operate, with the complex functioning as a lipophilic vehicle that delivers copper to the intracellular redox environment. The stability of the complex in biological media was not experimentally assessed in the present study; discriminating between these pathways would require speciation and stability studies under physiological conditions (*e.g.*, UV-Vis and electronic paramagnetic resonance monitoring), activity comparisons with the free Cu^2+^ salt and the free ligand, and copper-chelation assays (*e.g.*, bathocuproine or tetrathiomolybdate). These experiments can be carried out in future work.

## 4 Conclusions

This study presents a comprehensive characterization of the [Cu(theo)(H_2_O)_2_Cl_2_] coordination complex through an integrated experimental-computational approach. Structural analysis confirmed a triclinic crystal system with pentacoordinate Cu(ii) in a square-pyramidal geometry, exhibiting thermal stability up to 358 K. The dehydration enthalpy of 89.5 kJ mol^−1^ per coordinated H_2_O molecule significantly exceeds that of bulk water, reflecting the strength of metal–ligand interactions within the coordination sphere.

The combination of vibrational spectroscopy (FT-IR and Raman) with DFT calculations provided complete vibrational mode assignments, with excellent correlation between experimental observations and theoretical predictions in ethanol and water solvation environments. The calculated frontier molecular orbital properties, including HOMO–LUMO gaps (3.8 eV) and electrophilicity indices (6.8 eV), indicate adequate chemical stability and favorable reactivity for biological interactions. Molecular electrostatic potential mapping identified electronegative regions associated with carbonyl and coordinated water oxygen atoms as potential sites for biomolecular recognition.

Molecular docking studies suggested strong intercalative binding to DNA (*K*_i_ = 5.85 µM) and moderate interaction with BSA (*K*_i_ = 40.78 µM), consistent with the complex's molecular geometry and electronic properties. Most importantly, cytotoxicity assays demonstrated promising anticancer activity against three tumor cell lines (PC-3, MDA-MB-231, HCT-116) with IC_50_ values in the low micromolar range (2.91–3.77 µM), while exhibiting significantly lower toxicity toward healthy GM07429A cells (IC_50_ = 12.18 µM). The selectivity indices (SI > 1) for all cancer cell lines, particularly for MDA-MB-231 breast cancer (SI = 4.19), indicate preferential cytotoxicity toward malignant cells, a crucial requirement for therapeutic development.

The favorable biological profile of [Cu(theo)(H_2_O)_2_Cl_2_], combining potent cytotoxic activity against cancer cells with relative safety toward normal cells, positions this complex as a promising candidate for anticancer drug development. The dose-dependent response and superior selectivity compared to healthy tissue suggest potential advantages over conventional platinum-based chemotherapeutics. This work contributes to the growing field of copper-based complexes and demonstrates how rational design combining bioactive organic ligands with transition metals can yield complexes with enhanced therapeutic potential.

## Author contributions

Francisco N. B. Domingos: conceptualization, methodology, investigation, visualization, writing – original draft. João G. de Oliveira Neto and Jéssica A. O. Rodrigues: conceptualization, methodology, formal analysis, data curation, writing – review and editing. Jailton R. Viana: methodology, software, writing – original draft. Kamila R. Abreu: conceptualization, methodology, investigation. Aramys S. dos Reis: validation, investigation, writing – original draft. Mateus R. Lage: methodology, software, writing – review and editing. Francisco F. de Sousa: validation, investigation, writing – review and editing. Eliana B. Souto: validation, investigation, writing – review and editing. Adenilson O. dos Santos: conceptualization, validation, resources, writing – review and editing, supervision, project administration, funding acquisition.

## Conflicts of interest

The authors declare that they have no known competing financial interests or personal relationships that could have appeared to influence the work reported in this paper.

## Data Availability

Data will be made available on request.
